# A Two-Person Neuroscience Approach for Social Anxiety: A Paradigm With Interbrain Synchrony and Neurofeedback

**DOI:** 10.3389/fpsyg.2021.568921

**Published:** 2022-01-14

**Authors:** Marcia A. Saul, Xun He, Stuart Black, Fred Charles

**Affiliations:** ^1^Faculty of Media and Communication, Centre for Digital Entertainment, Bournemouth University, Poole, United Kingdom; ^2^Department of Psychology, Faculty of Science and Technology, Bournemouth University, Poole, United Kingdom; ^3^Applied Neuroscience Solutions Ltd., Frimley Green, United Kingdom; ^4^Department of Creative Technology, Faculty of Science and Technology, Bournemouth University, Poole, United Kingdom

**Keywords:** social anxiety disorder (SAD), hyperscanning, interbrain synchrony, neurofeedback, two-person neuroscience

## Abstract

Social anxiety disorder has been widely recognised as one of the most commonly diagnosed mental disorders. Individuals with social anxiety disorder experience difficulties during social interactions that are essential in the regular functioning of daily routines; perpetually motivating research into the aetiology, maintenance and treatment methods. Traditionally, social and clinical neuroscience studies incorporated protocols testing one participant at a time. However, it has been recently suggested that such protocols are unable to directly assess social interaction performance, which can be revealed by testing multiple individuals simultaneously. The principle of two-person neuroscience highlights the interpersonal aspect of social interactions that observes behaviour and brain activity from both (or all) constituents of the interaction, rather than analysing on an individual level or an individual observation of a social situation. Therefore, two-person neuroscience could be a promising direction for assessment and intervention of the social anxiety disorder. In this paper, we propose a novel paradigm which integrates two-person neuroscience in a neurofeedback protocol. Neurofeedback and interbrain synchrony, a branch of two-person neuroscience, are discussed in their own capacities for their relationship with social anxiety disorder and relevance to the paradigm. The newly proposed paradigm sets out to assess the social interaction performance using interbrain synchrony between interacting individuals, and to employ a multi-user neurofeedback protocol for intervention of the social anxiety.

## 1. Introduction

Social interactions are an integral part of our daily lives, from simple friendly gestures to group decision-making that may have great impact on each individual's lives. Undoubtedly, humans rely greatly on a peripheral support system, such as family or friends, due to an evolutionary instinct of survival through social mechanisms (Dunbar et al., 2007; Tomasello et al., 2012). As we have built a society driven by our advanced social abilities, the effect of adverse biological selection or mutation on the brain areas associated with these skills can bring about monumental disruptions to a person's quality of life.

In the past, social neuroscience studies have been conducted to explore the neural correlates of behaviour in response to social stimuli from a single individual (Ochsner, 2004). A rising concept known as two-person neuroscience (Hasson et al., 2012; Pfeiffer et al., 2013; Schilbach et al., 2013) (2-PNS) suggests that it may be more prudent to extend experimental protocol of social neuroscience to include neuronal activity from of all participating bodies in the interaction. Furthermore, the individuals involved in an interaction should be considered a functional unit for which to obtain the neuronal information regarding the social interaction which occurs within the unit. Typically, a functional unit known as a “dyad” contains two individuals. The dyadic setup, commonly known as hyperscanning (Montague et al., 2002; Babiloni and Astolfi, 2014), has facilitated 2-PNS research, most prominent of which is interbrain synchrony (InBS). Hyperscanning is the simultaneous brain scanning of multiple participants while allowing them to either interact with each other in an experiment or undergo tasks separately (Babiloni and Astolfi, 2014). This branch of research is concerned with understanding how neural synchrony, proposed between two or more brains, can occur and why. Recent InBS studies have found that weaker synchronisations occur when one member of a dyad reported having previously suffered anxiety (Kawano et al., 2016) or if members of the dyad were strangers compared to acquaintances (Atzil et al., 2012; Kinreich et al., 2017). 2-PNS argues that, particularly for social dynamics, the whole picture can be better represented by the findings from all interacting individuals (Garćıa and Ibáñez, 2014). Further research would dictate whether or not a relationship exists between InBS and mental health disorders that are characterised by social deficits. Moreover, the dyadic activity which occurs between the interactors may provide valuable insights to human social neuroscience. For that reason, it is necessary that investigations are conducted in the prospect of potentially improving current methods of treatment for mental disorders by applying knowledge gained from InBS or 2-PNS.

To understand the role of InBS, a novel paradigm implementing the InBS protocol into a contemporary treatment of social anxiety disorder is proposed. The differences between the paradigm and treatment alone would uncover factors of the relationship between InBS and social anxiety disorder. Neurofeedback is a viable contender for such a strategy, having potential to gain from the 2-PNS approach itself. The neurofeedback method is a non-invasive treatment for mental health disorders. It uses brain imaging techniques to capture real-time data on brain activities and acts as stimuli back to users. While InBS observes the modulation of neural synchrony between brains, neurofeedback aims to modulate the neural activity within each brain. In common practice, neurofeedback protocols are shaped around a single individual. However, the idea of multi-user neurofeedback has gained traction in the form of between brain interpersonal interaction without the conventional ways of communicating, such as verbal or physical (Duan et al., 2013; Kovacevic et al., 2015). As such, there exists a gap in the literature for the exploration of a multi-user based approach for the training or treatment of the mental system using neurofeedback.

This review aims to justify how essential the InBS-neurofeedback (InBS-NF) paradigm is for the impact potential on the current scientific capacity of social anxiety disorder. The remainder of this review is organised into the following sections: Section 2 defines social anxiety disorder by way of research findings from different neuroimaging methods. Section 3 reports the concepts and approaches for the paradigm components. Section 4 describes the paradigm with corroboration from 2-PNS literature and Section 5 concludes the review.

## 2. Social Anxiety Disorder

Social anxiety disorder (SAD) is a type of phobia induced by one or more social situations, causing an individual to feel inner turmoil (Stein and Stein, 2008). The disorder was differentiated from general anxiety over 50 years ago (Marks and Gelder, 1966) and is widely recorded as the most common anxiety disorder in present day (Stein et al., 2017), while being the third most common disorder overall (Kim and Yoon, 2018). The behavioural characteristics of SAD can be observed as evasive natures towards a person, a group of people or a specific type of situation which involves other people (Stein and Stein, 2008). The onset of an episode can cause physical manifestations which include trouble with breathing or speech, feelings of pain, tremors, uncontrollable crying, feelings of nausea, or excessive sweating (Agha Mohammad Hasani et al., 2016; Spence and Rapee, 2016). Commonly, the behaviour stems from a fear response of being negatively scrutinised or humiliated by or in front of people (Kessler et al., 1998). SAD has been found to have a negative impact on academic performance in children and work performance in adults, while increasing unemployment rates as a whole (Dryman et al., 2016). Additionally, data analysis from hospital databases indicates that not only is there a heightened likelihood for substance abuse, but also an increased mortality rate, particularly an increased co-morbidity rate when coupled with depression (Meier et al., 2016; Jarallah et al., 2017). Research has suggested a number of factors that contribute to the aetiology of SAD. Genetic predispositions (Arnold et al., 2004), biases developed in early childhood (Kuo et al., 2011), negative experiences (Kent and Keohane, 2001) and family/peer relationships (Johnson et al., 2001; Cunha et al., 2008) are often said to be important in understanding the individual development of SAD (Wong and Rapee, 2016).

Cognitive behavioural models of SAD have been put forward with the most commonly cited being the Clark and Wells (1995) model and the Rapee and Heimberg (1997) model (Morrison and Heimberg, 2013). Both models constitute a theoretical framework to understanding the development and maintenance of SAD in an individual. The Clark and Wells model posits that individuals with SAD enter a social situation holding cognitive biases and conditional beliefs. These cognitive biases include an unusually high performance for a successful social interaction, creating a high standard which is difficult to achieve and therefore an increased perception that they will fail at the social interaction. These conditional beliefs infer stringent rules (Penney and Abbott, 2014) that a specific action on the individual's part will lead to a negative reaction from the audience or interactor. Due to this, when entering a social situation, an individual's attention is shifted towards themselves—a process known as self-focused attention. The self-focused attention in turn activates further dysfunctional biases and beliefs manifesting in physiological outputs such as excessive sweating. These outputs are identified and processed in the self-focused attention model to generate a negative mental self-representation of themselves and are prevented from allocating attention to positive cues from the audience or interactor. Moreover, the Clark and Wells model proposes that individuals with SAD may use safety-seeking behaviours which contribute to the maintenance of SAD. For example, individuals with SAD may avoid a social situation and believe that safety was obtained through avoidance rather than the social situation being less threatening than perceived (Penney and Abbott, 2014). Equally, the avoidance being noticed by the audience may cause the individual to feel further scrutiny. Furthermore, safety-seeking behaviours allocate attention even more so towards an individual's self, exhausting cognitive capacity for acknowledging positive cues from the audience or objective information of the situation.

The model proposed by Rapee and Heimberg is theoretically similar to the Clark and Wells model, with the difference lying in a significant importance for positive judgement from the audience or interactor in the social situation. Therefore, the attentional shift is allocated to both the perception of self and external cues from the audience. Many of the same concepts in the self-focused attention model are also applied, whereby the individual generates a representation of themselves based on their own physiological reaction. Though, in addition to this, the Rapee and Heimberg model also suggests memories and the external cues from the audience contribute to this representation. Thus, a discrepancy is created between the representation of the individual's self and the perceived standard from the audience. The greater the discrepancy, the greater the individual with SAD believes a negative outcome from the social interaction will occur manifesting in the individual's physiology (such as feeling flustered) and behaviour (such as safety-seeking, escaping) and therefore maintaining SAD.

Treatment for SAD typically falls under two categories: psycho-social and pharmacological. The most common psycho-social treatment is cognitive behavioural therapy (CBT) while pharmacological treatment includes, but are not limited to, the use of antidepressants, benzodiazepines, and beta-blockers. Occasionally, other novel pharmacological treatments may be used if the general options are ineffective. It is also possible to combine psycho-social and pharmacological treatments. Reviews on the state of affairs for SAD treatment have corroborated that there is a lack of understanding for why certain treatments work better than others (Rodebaugh et al., 2004; Dalrymple, 2012) and why some of the best treatments are not capable of working at all (Liebowitz et al., 1999). 2-PNS suggests that these shortcomings of SAD treatment may be explained by investigating the interpersonal effects of social interaction, and that a fundamental aspect is being overlooked by focusing solely on the intrapersonal scale (Konvalinka and Roepstorff, 2012) where psycho-social and pharmacological treatments are bound. In this light, the InBS-NF paradigm is necessary to provide insight to new knowledge or information to improve SAD treatment by implementing the interpersonal protocol.

### 2.1. The Neuroscience of Social Anxiety Disorder

Through neuroimaging techniques, the complex neurobiological processes such as abnormal neural activity or dysfunctional connectivity that have been associated to SAD (Klumpp et al., 2012) are capable of being observed in research to determine the neural substrates of SAD. These neuroimaging techniques include functional magnetic resonance imaging (fMRI), functional near-infrared spectroscopy (fNIRS) and electroencephalography (EEG).

fMRI indirectly obtains information about neural activity by observing the blood-oxygen-level dependent (BOLD) signal which is based on the haemodynamic response. The haemodynamic response refers to an increased delivery of oxygenated blood to areas of the brain activated by a behaviour, in order to provide oxygen more than required for the brain region to function. This excessive amount of oxygen subsequently increases the oxygenated blood ratio relative to the deoxygenated blood. Oxygenated blood has magnetic properties which generates less interference with the signal when picked up by the fMRI machine compared to that of deoxygenated blood. Thus, an image can be drawn when an influx of oxygenated blood occurs over a specific area of the brain following a particular stimulus as oxygenated blood will provide a stronger signal whereas the more deoxygenated blood there is, the less the signal can be picked up by the fMRI scanner (Buxton et al., 2004). The fMRI technique allows for extracting information of neural activity that requires superior spatial resolution which is limited for both the fNIRS and EEG techniques (Cui et al., 2011; Kaiboriboon et al., 2012). We are able to observe changes within the subcortical levels of the brain and pinpoint the origin of these changes. However, a common concern of fMRI is low temporal resolution and limited degree of mobility. One of the central themes of fMRI research for SAD is the role of the amygdala. The amygdala is a subcortical structure, renowned for emotional processing and fear responses. Research suggests that imbalances in neural activity relating to SAD can originate from the amygdala and also be the result of the amygdala processing abnormal information from cortical areas (Liao et al., 2010). In anatomical and functional terms, the amygdala has been shown to have strong connections with the prefrontal cortex (Barbas, 2000; Bechara et al., 2000; Rule et al., 2002; Phillips et al., 2008). In particular, the orbitofrontal cortex (Liao et al., 2010; Hahn et al., 2011), the medial prefrontal cortex (Qiu et al., 2011; Sladky et al., 2012) and pathways between the frontal and visual lobes (Liao et al., 2010) have all exhibited activation relationships with the amygdala that can be attributed to SAD. The frontal-visual-cortex pathways have also displayed decreased connectivity during resting-state fMRI. For example, decreased connectivity has been shown to be correlated with the severity of SAD symptoms (Ding et al., 2011). In addition, resting-state fMRI studies with SAD have revealed several abnormal connectivities (Liao et al., 2010; Hahn et al., 2011) within frontal lobe regions (Ding et al., 2011). Furthermore, it has been suggested that significant abnormal activity in the posterior cingulate cortex and the precuneus in participants with SAD may reflect the impairment of socially relevant self-focused attention and theory of mind respectively (Gentili et al., 2009; Hahn et al., 2011; Nakao et al., 2011; Qiu et al., 2011). In addition to connectivity, fMRI studies have also guided the understanding of dyfunctional localisation of SAD. The most common report of dysfunctional localisation relating to SAD is hyper-reactivity within the amygdala and insula regions. Studies have shown that hyper-reactivity in this region is significantly present in individuals with SAD compared to healthy controls (Shah et al., 2009; Klumpp et al., 2010, 2013). Additionally, the extent of hyper-reactivity in the amygdala has been shown to have a relationship with the severity of SAD while the extent of hyperactivity in the insula correlates with trait anxiety (Shah et al., 2009). It is further suggested that amygdala hyper-reactivity can be triggered with moderate, even subtle, displays of social threat (Klumpp et al., 2010). In a longitudinal approach to SAD research, studies into the neural predictors of CBT have shown that activation within certain regions of the brain prior to treatment are positively correlated to the level of success after a 12-week period (Klumpp et al., 2013). Specifically, pre-treatment activation to socially negative stimuli within the superior and middle temporal gyrus, which play a role in visual processing, dorsal anterior cingulate cortex and dorsomedial prefrontal cortex, which play roles in cognitive and emotional processing, are observed within positive outcomes from CBT (Klumpp et al., 2013). fMRI imaging has been used to map a “skeleton” of networks across brain areas for information processing and cognitive functioning (Bressler and Menon, 2010). One of these networks is known as the default mode network, which is activated during periods of time where an individual is not focused on the outside world (Voelcker-Rehage et al., 2016). SAD is correlated with abnormal activity within the bilateral angular gyrus and the left prefrontal medial cortex when the default mode network is engaged (Qiu et al., 2011). Research suggests that these findings are related to impairments in social perception and the mental representation of self which contribute to the self-attentional bias model (Qiu et al., 2011). The pathway which is activated during high cognitive functioning behaviours such as memory retention, problem-solving and decision making is known as the central executive network (Petrides, 2005; Menon, 2011). This network shows abnormal activity within the right dorsolateral prefrontal cortex, right inferior parietal gyrus and the left middle occipital gyrus in SAD patients, reflecting an impairment of cognitive control, hypervigilance and hyperprosexia from individuals with SAD (Qiu et al., 2011).

The fNIRS neuroimaging technique operates using near-infrared light (typically 700–900nm spectral interval) that propagates through the brain to provide information up to 8cm deep through optodes. fNIRS is similar to EEG whereby it is non-invasive and has a high degree of mobility. On the other hand, it is similar to fMRI in that it works under the BOLD signal principles and haemodynamic response, which gives poor temporal resolution. The results from fNIRS research into neural substrates of SAD have recurred a frontal lobe theme. For example, frontal asymmetry has been found to occur with stronger signals in the right hemisphere by observing an increase of blood volume concentration when participants were asked to take part in an anxiogenic task (Tuscan et al., 2013). Additionally, significantly smaller changes in haemodynamic response during performance-based tasks in the ventrolateral and dorsolateral prefrontal cortex was reported from experiments with SAD diagnosed participants (Yokoyama et al., 2015; Glassman et al., 2017). The ventrolateral and dorsolateral prefrontal cortex areas are both known to elicit hyperactivity with SAD (Tillfors et al., 2002; Yokoyama et al., 2015), while also showing activation relationships with certain limbic structures (Guyer et al., 2008; Blair et al., 2010) such as the amygdala (Sladky et al., 2012). These findings suggest that the ventrolateral and dorsolateral prefrontal cortices illustrate a regulation or connectivity dysfunction between the frontal lobes and limbic structures.

EEG is a neuroimaging technique which measures the electrical activity in the upper layers of the brain by placing electrodes across the scalp. The major advantages of EEG recording are high temporal resolution and a high degree of mobility. EEG is able to record in order of milliseconds with sampling rates between 250 and 20,000 Hz. It is also relatively resistant to movement artefacts (Croft and Barry, 2000; Gwin et al., 2010) which can occur when the participant undergoes an experiment that requires mobility or if the participant is generally fidgeting. Additionally, while the fNIRS and fMRI methods are effective in their own right; they capture essentially metabolic processes which, at best, can be only associated with neural activity. EEG, on the other hand, is capable of measuring real neural responses. This provides us with great timing accuracy for identifying neural activity corresponding to external stimuli. Data recorded by EEG reveals the electrical activity from groups of neurons being recorded at each electrode which covers a specific area of the brain. These activities are analysed either in the time domain or the frequency domain. Using the time domain, the EEG signal is displayed as a continuous signal changing over time (Hjorth, 1970). The frequency domain, on the other hand, displays how much of the signal lies in a particular frequency band given multiple frequencies at hand (Valdés et al., 1992). EEG frequency bands are commonly grouped into seven different types of oscillatory activity. Firstly, delta waves (0.5–4 Hz) are the slowest and typically demonstrate the characteristics of an individual in deep sleep. Theta waves (3–7 Hz) are also related to oscillatory activity during sleep, however these are more prominent in the first stages of sleep or “light sleep”. Thus, typically appearing during meditative or drowsy states and in some cases during hypnosis. Alpha waves (8–12 Hz) are largely associated with relaxed, calm, alert and creative moods (Evans and Abarbanel, 1999) and predominantly occur within the occipital lobe when an individual is in a relaxed state with their eyes closed. When an individual has their eyes open, or is sleepy, alpha is decreased. Mu waves (9–11 Hz) are an alpha-like variant and is most prominent when the body is at rest and can be observed over the motor cortex (Hobson and Bishop, 2017). These waves are suppressed when an individual performs or visualises a motor action. Beta waves (13–29 Hz) are commonly observed as indicators for mental performance, including levels of focus, concentration, active thinking and problem-solving (Herrmann et al., 2016). Gamma waves (30–40 Hz) are the highest and show associations with higher cognitive functioning, including being related to large scale brain network activities including memory, attention and perception (Herrmann et al., 2016). However, gamma wave frequencies can overlap with electromyography (electrical activity produced by skeletal muscles) frequencies and proper separation during signal processing is necessary to ensure the source of the signal (Muthukumaraswamy, 2013). Each of these frequency bands have associations with certain types of neurophysiologies and behaviours, and therefore activities which strays from the norm can be useful to deduce the cause of abnormal behaviours.

EEG affiliation with SAD predominantly include research focusing on the frontal lobe of the brain, measuring frontal asymmetry and delta-beta cross frequency correlation (Harrewijn et al., 2016). Frontal asymmetry was proposed on the theory that the left and right areas of the frontal lobe are differentially involved in positive and negative affective states (Davidson, 2000). Experiences and feelings of a positive affect are processed within areas of the left frontal lobe while the negatives are processed within areas of the right frontal lobe (Moscovitch et al., 2011). Specifically, individuals with SAD or those who experience symptoms of SAD have been shown to display significantly stronger activations in the right frontal lobe compared to the left during both resting states and anxiety-induced tasks (Davidson et al., 2000; Beaton et al., 2008; Moscovitch et al., 2011). In addition, activity elevations in the right frontal lobe has been found in individuals who display a bias towards withdrawal-related behaviour, compared to the left that are biassed towards approach-related behaviour (Davidson, 1992, 1998). This theory has been corroborated by several studies over the past decades (Schmidt, 1999; Coan and Allen, 2004; Campbell et al., 2007; Hannesdóttir et al., 2010). Secondly, delta-beta cross-frequency correlation has also been a topical approach in literature for understanding neural substrates of SAD. Cross-frequency correlation is a type of cross-frequency coupling metric which is formed on the basis that brain oscillations of varying frequencies are capable of interacting with each other. This type of interaction can be observed during complex information transmissions between high-frequency activity in local, cortical regions and low-frequency activity which stem from a larger, regional network (Salimpour and Anderson, 2019). When this interaction involves the coupling between a phase of an oscillation from one frequency band and the amplitude of the oscillations from another frequency band, this is known as phase-amplitude, or phase-power, coupling (Jirsa and Müller, 2013; Salimpour and Anderson, 2019). Other forms of cross-frequency coupling include phase-phase and amplitude-amplitude coupling (Jirsa and Müller, 2013). A positive amplitude-amplitude delta-beta cross-frequency correlation is known to increase with anxiety-induced social tasks (Schutter and Knyazev, 2012). Research also suggests that the oscillations which fall within the delta band are in fact neural activities which originate from sub-cortical regions of the brain whereas beta oscillations originate from the cortical regions (Schutter and Van Honk, 2005). And therefore, the cross-frequency correlation is an indicator of the communication between sub-cortical and cortical regions (Harrewijn et al., 2016). Increased positive delta-beta cross-frequency correlation has been observed in studies with individuals diagnosed with SAD (Miskovic et al., 2011a,b; Harrewijn et al., 2016) and individuals with high levels of reported feelings or emotions symptomatic of SAD (Miskovic et al., 2010).

EEG, fNIRS and fMRI methods have contributed collaboratively in SAD research to help understand the aetiology and maintenance of SAD on a neurological scale. Each method is capable of extracting useful information from the brain which link together to provide a full picture. InBS and neurofeedback research have both utilised demonstrated the use of varying neuroimaging techniques depending on the task and level of exploration involved. For the InBS-NF paradigm, the main conceptual premise relies on an interactable, naturalistic environment with a capacity for mobility. In addition, the synchronicity calculations and feedback latencies advocates for a higher temporal resolution to increase accuracy. The downfall of the fMRI scanner, on top of the has reduced flexibility for mobility and requires participants to lie in a large and loud scanner typically on their own. Additionally, EEG has superior temporal resolution in comparison to the fNIRS approach. Due to this, the InBS-NF paradigm leans towards EEG as the neuroimaging protocol. EEG maximises the ability for participants to interact with each other and real-time feedback stimuli during an experimental task while limiting the movement artefacts.

## 3. The Paradigm Components

As the term suggests, the InBS-NF paradigm proposed by the authors is a method which integrates two components; namely the information of synchronicity between interactors and the existing neurofeedback protocol. The aim of InBS-NF is to utilise the measure of InBS to constantly assess SAD in an objective manner, and to employ active neurofeedback to directly manage SAD. Subsequent work to this review include exploring the role of InBS and SAD across neuroimaging techniques to provide a full picture, spatially and temporally, of the interbrain activity occurring during socially anxiogenic tasks. Additionally, a foundation of research utilsing the InBS-NF paradigm is required for empirical investigation to determine the effect of multi-participant neurofeedback using InBS on individuals with SAD. In sight of the limited research incorporating InBS and SAD, the InBS component will be detailed in this section by way of the conceptual frameworks involving interactions that have been previously shown to affect individuals with SAD. Similarly, the neurofeedback component is discussed with approaches prominently used in literature to research the relationship between neurofeedback treatment and SAD.

### 3.1. Interbrain Synchrony and Interaction Frameworks

InBS describes how similar the oscillations of membrane potentials, or frequencies, are between two networks of neurons. More specifically, the signals between the individuals in a functional unit during an interaction (Babiloni et al., 2006). Synchronicity can be observed when these signals are both time-locked and phase-locked (Lachaux et al., 1999). For signals to be time-locked, they must display similar oscillations of frequencies at roughly the same time post-stimulus onset. For phase-locking, the signals must have the same phase angle as each other. InBS is analogous to brain-to-brain coupling of neural activities from regions or networks between brains (Gonçalves, 2015), which can occur between different frequency ranges. These couplings do indeed share certain aspects, though ultimately differs to cross-frequency coupling in that the measurements are observed and analysed from two separate brains in comparison to a single brain in cross-frequency coupling research.

Interbrain analysis began dubiously as “extrasensory” communication between the brains of a set of twins (Duane and Behrendt, 1965). While the authors stated that no conclusive statements were made from the findings of this study, their study reports that by increasing alpha wave oscillations in one twin, the other twin's brain would mimic these activities. The reception of these findings were greatly sceptical, due to the lack of evidence that the change in one participant did indeed elicit the change in the other (without any form of interaction). During that period of time, EEG neuroimaging techniques were in its infancy and the technology was not available to produce reliable results on neural correlates of social behaviour. In fact, it took another 40 years for the field to introduce the first hyperscanning protocol using fMRI to compliment single-participant social neuroscience studies (Montague et al., 2002). This protocol erupted multi-participant social neuroscience research (Babiloni and Astolfi, 2014). Hence, although the understanding of the social aspect in human nature has been thoroughly evidenced, limitations in technology meant that only in the past couple of decades has investigations on the neuroscience of social interactions been done (Babiloni and Astolfi, 2014). Furthermore, the first implementation of hyperscanning using EEG was conducted shortly after the protocol debut (Babiloni et al., 2006), providing the space for better temporal resolution and ecological validity (Babiloni et al., 2006). Eventually, in accordance with refined procedures and technological feasibility, empirical InBS research came to fruition using the hyperscanning protocol (Dumas et al., 2010).

#### 3.1.1. Physical Space

Research has shown that InBS is observed when interactors share and interact within the same physical space. For example, joint action, which can involve two or more people, refers to the process of co-ordination between the actions of the interactors in time and space to produce a joint outcome (Knoblich et al., 2011). Many of the joint action interactions in InBS research fall in line under sensorimotor co-ordination, such as the leader-follower task model—a construct where members of a dyad adopt social roles in the experiment. In experiments implementing leader-follower dynamics with guitar playing, research has shown interbrain synchronisation in theta and delta waves between the frontal and central sites of the interacting brains (Lindenberger et al., 2009; Sänger et al., 2012). Movement and speech co-ordination tasks evokes interbrain synchronisation of the alpha and theta bands over the temporal and lateral-parietal brain regions (Kawasaki et al., 2013). Implicit co-ordination in timing has also been related to increased brainwave synchrony when the timing discrepancy within the dyad decreased (Funane et al., 2011). Significant differences were reported between the synchronisations between the leader-follower task model and a follower-follower model (Jiang et al., 2015); greater InBS occured within the leader-follower task when the synchronisations were directed to the follower from the leader.

Another type of physical space interaction associated with InBS is affective communication. This refers to both the expression of one's internal emotional state regarding another person or situation, and the perception of another individual's or group's signals regarding their emotional state. Examples of affective communication during an interaction include emotional interchange, support or empathetical expression (Viswesvaran et al., 1999; Symons et al., 2016). Research found higher levels of InBS over the left inferior frontal cortex while a dyad communicated face-to-face with each other in comparison to not (Jiang et al., 2012). This effect over the same brain areas also transpired when dyads hummed facing each other rather than facing walls (Osaka et al., 2015). A classroom setting experiment found that enhanced alpha synchrony between students was present when they sat face-to-face in comparison to adjacent. This result postulates an indicator role of InBS to predict the engagement of students and their potential social dynamics (Dikker et al., 2017). Furthermore, a study measured synchrony from a co-operative task within a dyad before and after undergoing co-operative tasks with a PC (Kawasaki et al., 2013). The findings showed greater synchrony in the alpha and theta frequency bands between the temporal and lateral-parietal areas within the dyad after the PC condition compared to before. The authors speculated that this is due to a lack of empathy perception during the machine condition in between the human-human conditions, which influences the increase in synchronicity due to a lack of or decrease in empathy perception during the machine condition.

Decision-making tasks also constitute a physical space interactive framework for InBS. These consist predominantly of behavioural studies in economic reasoning through game theory. The general consensus of findings revealed that co-operative behaviours induced a greater level of InBS than competitive or defective through the prisoner's dilemma task (Babiloni et al., 2007; Astolfi et al., 2009; Jahng et al., 2017); similarly with joint vs. solo tasks (greater InBS with joint tasks compared to solo) (Astolfi et al., 2014; Sinha et al., 2016). In addition, flight simulations with pilots and co-pilots while simultaneously examining their brainwave patterns (Astolfi et al., 2011; Toppi et al., 2016) found that there was a significantly increased level of alpha band synchrony over parietal sites and increased synchrony over frontal sites. During the phases of flight simulation where interaction was peaked (i.e., co-cooperativeness during take-off and landing), an enhanced synchronisation over the frontal regions of both participants was observed. By using the partial directed coherence measure, they were able to deduce that this synchronisation was directed towards the pilot originating from the co-pilot. Synchrony analysis techniques, including the partial directed coherence, are briefly discussed in section 4. Moreover, a button-pressing experiment for the quickest reaction time using both co-operative and competitive conditions was conducted (Cui et al., 2012). Co-operation trials displayed significantly greater synchrony in the superior frontal cortex compared to competition. InBS during interactive decision-making tasks shed light on synchronicity as an indicator for social interactions or co-operative task performance (Cui et al., 2012). Its role as a valence indicator was put forward when significantly lower levels of synchronisation occurred while both participants in a prisoner's dilemma game set out to implicate their partner and higher levels if both participants intended not to Fallani et al. (2010). Additionally, specific brain activities during InBS prior to decision making during the task was able to predict the subsequent action with up to 90% accuracy (Fallani et al., 2010).

The above interactions that exhibit InBS correlates have inherent links with different levels of SAD. The extent of affective communication tasks posits potential avenues for understanding the effect of InBS on interpersonal functioning (Gonçalves, 2015), in particular with participants who report struggles with social situations. Affective processing of social exclusion showed that participants with SAD were significantly more likely to direct the attention internally, experiencing mental frameworks such as self-blame or rumination, than the healthy controls (Gutz et al., 2016). This finding ties into the self-focused attention component of the maintenance models of SAD (Clark and Wells, 1995; Rapee and Heimberg, 1997). InBS studies on affective communication has potential to uncover aspects of this increased self-focused attention by illustrating patterns in synchronicity while one interactor experiences symptoms of SAD. Economic exchange games, similar to that of the decision-making and game theory tasks above, have shown differences in brain activation between participants with SAD and healthy controls. For example, an fMRI study found significantly lower activation of the medial prefrontal cortex was observed when participants with SAD were asked to play a “trust game” to probe mentalizing of the participants (Sripada et al., 2009). Another socioeconomic task known as the “ultimatum game,” which asks participants to accept or reject offers from their interactor, found that participants with SAD were more likely to accept unfair offers than the healthy controls (Grecucci et al., 2013). These studies can be associated with safety-seeking behaviours such as avoidance (Maner and Schmidt, 2006) to avert likelihood of social confrontation (Hampel et al., 2011) or unpleasant social situations. Based on the evidence in literature, these interactions which evoke InBS are clearly associated with SAD.

#### 3.1.2. Mental Space

InBS has been associated to mental space synchronisations between interactors. For example, the shared attention framework describes the moments where two or more individuals are focused on, and are aware that the collective focus is on, a particular third entity (Shteynberg, 2015, 2018). The mutual focus on a third entity is often achieved by one individual in the interaction or a third party directing the attention towards the entity through verbal or non-verbal communication i.e., joint attention; (Baldwin, 1995; Tomasello and Akhtar, 1995). This is different to joint action where the interactors partake in a physical co-ordination of action. Experimental tasks to achieve shared attention in InBS studies largely involve non-verbal categories of communication. For instance, spontaneous and reciprocal gesture imitation has been found to elicit InBS during low-level motor processing and asynchrony during high-level processing (Dumas et al., 2011). During synchronous periods, the alpha-mu, beta and gamma bands between the centroparietal regions of both brains were reported to play key roles in social co-ordination (Tognoli et al., 2007). These findings reflect the different levels of information processing towards the higher-level motor processing and comprehension of social roles, in this case, the model or the imitator (Dumas et al., 2011). Traditionally, the term “joint attention” pertains to eye gaze following; frameworks which were also studied in InBS research to show significantly greater synchrony in alpha, betta and gamma bands between the prefrontal cortex and anterior cingulate cortex, compared to a control without eye gaze (Astolfi et al., 2010).

Joint attention is known to play an important role in the development of social skills, competence and engagement (Carpenter et al., 1998; Mundy and Gomes, 1998; Mundy and Acra, 2006). Studies have suggested that joint attention skills that were underdeveloped at even a very young age, such as 30 months, was capable of predicting social and emotional abilities later in life (Mundy and Sigman, 2006; Parlade et al., 2009). The development of joint attention between an infant and caregiver is a type of interaction which facilitates social learning (Mundy and Newell, 2007). For example, an infant learning the name of an object requires the infant to respond to the joint attention from the caregiver whose attention is on the object (Mundy and Newell, 2007). The lack of developed joint attention skills in childhood has often been associated to autism spectrum disorder (ASD) (Dawson et al., 2004), a disability that evidences partly overlapping symptoms with SAD i.e., gaze avoidance (Kleberg et al., 2017). An fMRI study reported a lack of InBS between the inferior frontal gyrus in individuals with ASD (Tanabe et al., 2012). They suggested that this lack of synchronicity was due to a struggle to share intention through eye contact. This finding was corroborated by another fMRI study which showed greater synchronisations in the inferior frontal gyrus when participants were able to see each other's eye gaze compared to when they were not (Saito et al., 2010). Therefore, the research suggests that there is indeed a link between interbrain synchronicity and joint attention, a mental form of interaction. A detriment of joint attention during early development in childhood could be linked to the levels of SAD that correspond to symptoms rising from a lack of social competence (Mundy and Sigman, 2006). Research into the relationship between InBS and SAD remains aspiring, along with the associations with other mental disorders. From the literature of social interactions that shows InBS, there is evidence of potential that these interactions and the corresponding findings of interpersonal neural synchronicity can contribute to aspects of the SAD model.

### 3.2. Neurofeedback

Neurofeedback originated in the 1960s as EEG biofeedback (Hammond, 2007), however, in modern days, neurofeedback has been accomplished in varying neuroimaging techniques alongside EEG such as fNIRS and fMRI. Neurofeedback has been researched and implemented as treatment for SAD, and the procedure involves:

- Acquisition of brainwave patterns
- Processing of acquired brainwave patterns to derive information
- Translation of this information into feedback that is fed back to the individual via sensory inputs

The purpose of this is to exploit latent plasticity for the enablement of improved brain regulation, brain functionality and enhancements on cognitive and behavioural abilities. Neurofeedback is based on two common assumptions: First, that the EEG activity (or activity deriving from the neuroimaging technique) has information about brain activities and that these activities have a bearing on the mental traits and state of the individual (Thompson and Thompson, 2003). Second, these mental traits and states can be altered as a result of providing feedback back to the brain based upon the EEG activity (Thompson and Thompson, 2003). Neurofeedback is typically used for individuals who show brain dysfunction (clinical use), however, it may also be used to achieve peak performance (non-clinical use). Notwithstanding these common features and the common basis, neurofeedback approaches are not homogenous. Traditionally the literature has classified the heterogeneity using the terms “active” and “passive”. Active neurofeedback describes a feedback process which incorporates the user's conscious decisions to make alterations on the amplitude of their brain activities (Cavazza et al., 2014; Jeunet et al., 2018). For example, modern assistive technology has utilised active neurofeedback in systems which allow brain-controlled movement of a wheelchair (Millán et al., 2009); the user imagines left- and right-hand movements (a process known as motor imagery) to send direction instructions to the wheelchair. Another utilisation of active neurofeedback involves a user modifying their frontal alpha asymmetry by responding to stimuli showing the current state of their frontal alpha rhythms, a measure shown to contribute in reducing negative affect and anxiety (Rosenfeld et al., 1995; Aranyi et al., 2016; Mennella et al., 2017). The main objective of active neurofeedback is to make use of the user's ability to achieve conscious control of their brainwaves and mental states, though such control requires both calibration of the neurofeedback system to the user and copious amounts of training effort from the user (Prpa and Pasquier, 2019). Passive neurofeedback, on the other hand, is opposite to active neurofeedback and does not explicitly involve the user's conscious commands to modifications of the user's brain activities (Prpa and Pasquier, 2019). This approach infers mental states from the continuous signal received by neuroimaging, which is then adapted to by the neurofeedback system employed (Jeunet et al., 2018). Passive neurofeedback is advocated by researchers because it allows for both the open monitoring of a user's brain activity that would be difficult to obtain and analyse with active neurofeedback (George and Lécuyer, 2010) and reduces the mental load of calibration and training for the user (Prpa and Pasquier, 2019). An example of passive neurofeedback is seen in affective computing, whereby the machine receives information of the user's emotional state and adapts the interaction with the user, with the option to also express affect in response to the user (Picard, 2000). Passive neurofeedback plays a role in adaptive automation (George and Lécuyer, 2010), detecting reduced arousal levels and alerting the user to increase concentration during a strenuous or high-risk task, such as driving. Ergo, the principal difference between the neurofeedback approaches is whether or not the neurofeedback system is responding to a user who is exerting conscious control over the activity of their brain rhythms to reach or maintain a certain pattern, or to a user who does not consciously attempt to modulate their brain rhythms but rather interact as normal or naturally with the system itself.

Another dimension of heterogeneity within the field of neurofeedback has been the degree of reliance on quantitative EEG (qEEG) techniques. qEEG is the numerical analysis of the characteristics of an EEG signal and the comparison of these characteristics with those of previously collected data. This previously collected data with which a comparison is made can be derived from a database of EEG signals, consisting of data from healthy individuals who show no brain dysfunctions (Kropotov, 2010), with the objective of identifying deviations from the calculated norm. Comparisons may also be conducted to identify patterns associated with particular mental traits or states, in which case, these are usually those associated with dysfunction (Kaiser, 2005; Kropotov, 2016). The resultant analysis is then translated into feedback, which is received and reacted upon by the individual undergoing neurofeedback to correct problematic patterns or encourage helpful patterns. In this process, an operant conditioning approach is applied. Common mental states targeted by neurofeedback include calm or relaxed vs. focus or concentrated (Chapin and Russell-Chapin, 2013), though the state of mind of an individual goes beyond these and lie in several paradigmatic states within beliefs and desires. The use of neurofeedback techniques can generally be regarded as one of two procedures: direct and indirect (Zander et al., 2010), with the latter sometimes being referred to as endogenous—meaning “from within” (Othmer et al., 2013). Both are used for clinical and non-clinical applications of neurofeedback.

#### 3.2.1. Direct vs. Indirect Neurofeedback

Direct neurofeedback incorporates an active approach, which seeks to change the EEG patterns (Thompson and Thompson, 2003) using the learning model of operant conditioning (Vernon et al., 2003), with an externally defined success criterion or criteria. This approach implies that the feedback portion is followed up by an active engagement of the individual in response to the neural activity that is being displayed to them (Thompson and Thompson, 2003) as the user is consciously aware of the changes that are occurring within their brains. This active engagement is steered using strategies which involve a success criterion (Aranyi et al., 2015), allowing the user to learn and exert a degree of control over which mental strategy returns positive feedback. Neurofeedback sets out to achieve modulation of neural activity by increasing or decreasing the power of a particular EEG frequency or across multiple frequency bands within a targeted brain area, with the goal to self-regulate behaviours or cognitive structures (Enriquez-Geppert et al., 2017). An individual undergoing neurofeedback is often asked to reach a particular mental state with the guidance of their own brain activity fed back to them during the exercise, typically in the form of auditory or visual feedback. As the individual practises to achieve the mental state, the power of a certain EEG band will be adjusted to the pattern of that mental state and the direction of this change is fed back to the individual. The feedback information will inform the individual whether the practise is working, and the individual may maintain the strategy or alter strategies to reach the desired mental state. This is an example of the feedback loop of neurofeedback where, once trained, alleviation of negative symptoms and enhanced regulatory patterns can be achieved using the mental strategies learnt during a neurofeedback session. One of the major benefits is that this type of self-regulation has the potential to be achieved independently of medication or psychotherapy (Hammond, 2007), where increased self-regulation has been shown to be synonymous with increased performance (Marzbani et al., 2016). The success of this strategy varies across individuals based on their own ability and initiative to utilise the skills learnt from treatment to “control” the EEG patterns (Quaedflieg et al., 2016). This aspect is particularly helpful, either on its own or in combination with medication, for individuals with SAD who would routinely find themselves in anxiety-provoking situations. It can also contribute to self-help or self-guided approaches to treatment for individuals who fall sub-threshold for diagnosis of SAD though still experience symptoms problematic enough to cause disruption in their lives (Dalrymple, 2012).

Indirect neurofeedback incorporates a passive approach, which does not have an externally defined success criterion/criteria. The brain is provided with a representation of its EEG activity via feedback and subsequently decides, unbeknownst to the individual, how to interpret this information. Indirect neurofeedback stems from empirical clinical work which concluded that the imposition of the operant conditioning model subjected the brain to less information than it could make use of Othmer and Othmer (2016). By giving the brain access to the signal without prescribing outcomes, on a continuous basis, and leaving the execution of the neurofeedback process to the discretion of the brain, this allowed the brain's natural proclivity toward self-optimisation to be executed with greater freedom and refinement (Othmer and Othmer, 2016). Indirect approaches to neurofeedback have emerged using infra-slow oscillations of the subcortical potential (Aladjalova, 1964). Infra-low frequency (ILF) neurofeedback targets the modulation of brain activity that lies below 0.5 Hz (Legarda et al., 2011), also known as infra-slow oscillations (ISOs). It has been suggested that the fluctuation of ISOs represent a fundamental component of regulation of brain functioning such as the modulation of cortical excitability (Lőrincz et al., 2009; Palva and Palva, 2012; Hiltunen et al., 2014; Grin-Yatsenko et al., 2018b). Additionally, ISOs have been proposed to derive from non-neuronal origins (Legarda et al., 2011) with relationships to ATP-derived adenosine (Lőrincz et al., 2009) and astrocytic oscillations (Parri and Crunelli, 2001; Parri et al., 2001). ILF neurofeedback methods incorporate the use of audio-visual feedback in real-time animations to provide feedback that the trainee is not able to volitionally follow (Grin-Yatsenko et al., 2018b). The mechanisms of ILF improvement are deemed to involve the re-normalisation of functional connectivity of resting state networks (Othmer et al., 2013). Research at the Russian Ministry of Health (Dobrushina et al., 2015) showed systemic changes in functional connectivity within the default mode network using functional magnetic resonance imaging (fMRI) data on a number of trainees after single sessions of ILF. ILF neurofeedback has been successfully implemented to treat traumatic brain injury, attention-deficit hyperactivity disorder, depression, anxiety and autism spectrum disorder (Othmer et al., 2013; Smith et al., 2014) with positive outcomes (Grin-Yatsenko et al., 2018b).

#### 3.2.2. Applications to Social Anxiety Disorder

While both the direct and non-direct neurofeedback techniques have previously exhibited beneficial outcomes (Raymond et al., 2005; Grin-Yatsenko et al., 2018a), further research is necessary for the application of both the techniques on SAD. Moreover, it would be useful to determine whether significant differences occur between directive and non-directive neurofeedback on the results for the effects on SAD and other mental disorders or peak performance. As neurofeedback gains traction in academic, medical and clinical practises, such further research benefits greatly on a standardised protocol for experimental design, allowing for the production of reliable results and comparisons to be drawn from Ros et al. (2020). Nevertheless, research into the applications of neurofeedback for the treatment of SAD have largely followed the direct route for training methodology.

One of the applications of neurofeedback to SAD is the alpha-theta protocol. The premise of alpha-theta was originated with the theory of accessing a hypnogogic (Gruzelier, 2009) state of mind auxiliary to deeper creative insights (Thompson and Thompson, 2003). When an individual is relaxed with eyes closed, alpha activity can be seen to increase towards a state of relaxation (Egner et al., 2002). As the individual begins to fall asleep, alpha activity decreases while theta becomes more dominant (Hasan and Broughton, 1994). This alpha-theta crossover in dominance normally occurs when an individual is in the first stage of the sleep cycle, whereas alpha-theta training teaches an individual to increase theta over alpha and therefore consciously access a mental state which is normally unconscious (Egner et al., 2002). By doing the training, an individual gains control over the low-frequency EEG activity to reach and maintain a state of deep relaxation (Raymond et al., 2005). Increasing the alpha-theta ratio (theta over alpha) at the parieto-occipital areas has been found to improve socially relevant anxiogenic mood states over a 5-week program with cumulatively progressive alpha-theta ratio over time (Raymond et al., 2005). Additionally, self-reports of social phobia measured with Social Phobia INventory (SPIN) score (Connor et al., 2000) significantly reduced over a 6-week program using the alpha-theta protocol, similarly over parieto-occipital areas, with an significant increase in the alpha-theta ratio over time compared to the no-neurofeedback group (Zhang and Cheng, 2017).

Another neurofeedback intervention for SAD is known as frontal asymmetry. As mentioned, in section 2.1, elevated relative right hemispheric activity is concurrent with SAD symptoms and observed in individuals with SAD. Neurofeedback applications to frontal alpha asymmetry entail to reduce this asymmetry between hemispheres. Alpha power is assumed to be the inverse index of neural activity in the cortex (Davidson et al., 1990; Cook et al., 1998). This means that the greater the alpha power, the less cortical activation is observed (Davidson et al., 2000; Oakes et al., 2004; Becker et al., 2015). Thus, alpha modulation of frontal asymmetry sets out to either reduce left hemispheric alpha or increase right hemispheric alpha (Allen et al., 2004), given that the approach-avoidance hypothesis suggests higher activation of the left frontal brain regions associated with approach and right frontal brain regions associated with avoidance (Davidson, 1992; Tomarkenand and Keener, 1998). However, it is important to note that imbalances can be driven from left, right or both frontal regions (Mennella et al., 2017) depending on the direction of affect dysregulation. For example, a dominance in avoidance behaviours can be characterised by an increase in relative right activation whereas a reduction in approach behaviours can be characterised by a decrease in relative left activation (Mennella et al., 2017). Affective BCI research has successfully exhibited interactions with virtual agents using frontal asymmetry with the approach-avoidance framework, whereby dorsolateral prefrontal cortex asymmetry directed towards a relative increase in left brain regions (approach) induces positively valenced responses to the user (Aranyi et al., 2015, 2016). Alpha modulation training, in particular, has obtained positive results for anxiety disorders. For instance, research showed that significantly reduced alpha asymmetry achieved improvements immediately after training (Spielberger, 1983; Mennella et al., 2017) and within 6 months afterwards (Kerson et al., 2009). Moreover, significantly decreased alpha asymmetry driven by lower alpha at the left-hemisphere sites was found with a coherent reduction in negative affect and anxiety symptoms, measured by the Positive and Negative Affect Schedule (PANAS; Watson and Clark, 1988) and the Beck Anxiety Inventory (BAI; Beck et al., 1988), following alpha modulation neurofeedback training (Mennella et al., 2017).

Lastly, research has shown neurofeedback applications for control of the amygdala. Neurofeedback training of the amygdala is frequently characterised by the top-down regulation of the prefrontal cortex. The amygdala and prefrontal cortex are both considered as important contributors in the circuitry of processing positive and negative affect (Davidson, 2000). Thus, the integrity of the pathways between the two regions plays in important role in regular cognitive functioning in social situations (Kim and Whalen, 2009). Self-regulation of anxiety was achieved using fMRI neurofeedback by providing participants with information of the activity of the ventrolateral prefrontal cortex. Levels of self-reported anxiety was negatively correlated with increased connectivity between the amygdala and ventrolateral prefrontal cortex post-training by neurofeedback (Zhao et al., 2019). Furthermore, neurofeedback training has also shown to effect resting-state fMRI through emotion self-regulation, specifically by increasing functional connectivity between the basolateral and centromedial amygdala with regions of the prefrontal cortex (Li et al., 2016). Increased functional connectivity between the amygdala and frontal lobe areas also demonstrated a correspondence with lower activations of the amygdala (Sarkheil et al., 2015; Herwig et al., 2019). Neurofeedback studies of amygdala self-regulation can provide a scientific grounding for the understanding that process of training itself would be effective with individuals diagnosed with SAD. fMRI research for amygdala activity can work together with EEG to provide EEG “fingerprints” (EFP) of amygdala activity (Meir-Hasson et al., 2014), empowering research to focus on the amygdala while individuals undergo tasks with greater ecological validity than that of fMRI. Such use of EFP has previously been implemented in amygdala-EFP neurofeedback with positive outcomes in the active downregulation of the amygdala-EFP signal during high-stress scenarios (Cohen et al., 2016; Goldway et al., 2019). However, there remains a gap in the literature for additional studies not only for amygdala regulation but also the prevalence of neurofeedback approaches to SAD.

## 4. The Two-Person Neuroscience Approach

Exploring the neural substrates of social behaviour have found that specific types of interactions trigger synchronous activities between brains. This interbrain interaction, according to the literature, seemingly cannot be observed from studies that test single participants (Krill and Platek, 2012; Chatel-Goldman et al., 2013; Schilbach et al., 2013; Koike et al., 2015). Previous research has shown that by using hyperscanning techniques and off-line analysis, significant differences in brain activities are present between brains related to synchronicity when performing tasks in collaboration or in isolation. For example, a dual n-back task (a test for working memory) using fNIRS found that InBS significantly increased in the prefrontal cortex during the paired condition where participants completed the task together compared to the single condition where they completed it alone (Dommer et al., 2012). Another fNIRS experiment involving comparisons of brain activity during a co-ordination task and an independent task also showed significantly increased synchrony in the frontal areas of the brain when participants co-ordinated rather than performing independently (Hu et al., 2017). Visual search tasks using EEG were carried out whereby participants were asked to attend to the task alone in one condition, and separately in another. The results found that greater interbrain synchronisations were present when the participants attended the same task (Szymanski et al., 2017) together compared with alone. Furthermore, EEG analysis from brain activity during a finger-tap matching experiment also showed greater synchronisations when participants matched patterns with another participant over the frontal and motor areas of the brain compared to when matching with a computer (Konvalinka et al., 2014).

These studies support the notion that brain activities recorded from one member of a dyad significantly differs when placed in a task with another person compared with being placed in a task on their own or with a robot (Ménoret et al., 2014). It can be observed that InBS is a promising measure of social interaction, beyond the aspects of social encounters that we can see or hear (Garćıa and Ibáñez, 2014). In this context, the importance of InBS lies in an intrinsic element of an affective component, namely the natural constitution of social exchange and psychophysiological connectivity (Coan et al., 2006; McAssey et al., 2013; Acquadro et al., 2016; Balconi and Vanutelli, 2016). Therefore, observed psychophysiological measures which occur between interactors indeed play a large role in understanding social interactions (Hari and Kujala, 2009; Konvalinka and Roepstorff, 2012). To emphasise, SAD is inherently a social interaction problem, which is based heavily on interpersonal phenomena. Therefore, InBS has a strong potential for quantifying interpersonal activity, which have typically been done on a single brain (Lahnakoski et al., 2014), to highlight aspects or levels of SAD that may not have been illuminated in studies consisting of a single participant.

### 4.1. An Auxillary to Existing Models

The InBS-NF paradigm incorporates the neural synchrony between interactors into a neurofeedback framework. In section 2, we describe two cognitive-behavioural models of SAD. Both models are based on an individual's perception and anticipation of an interaction (Norton and Abbott, 2016). Furthermore, the models are framed around individual reports of their own thought processes and how they feel or felt about other people (Norton and Abbott, 2016). Social neuroscience studies regularly used this one-person framework in experimental design, where mainly the participant's instruction was to either take part in an interaction or think about one (Schilbach et al., 2013). The core concepts that are based on individual processes of social cognition lay out the fundamental understandings of SAD. As an auxiliary, the InBS-NF paradigm offers a 2-PNS structure to existing SAD models, thus uses the 2-PNS notion to assist consolidating existing models by focusing specifically on how measures of the interpersonal aspect of interactions contribute to social deficits caused by SAD. This is particularly helpful for existing models that have an interaction component. For example, there is a stage in Rapee and Heimberg (1997) model that defines external cues as a contributing process to mental self-representation. These external cues are indicators which can be perceived as negative evaluation from the audience and inflate the discrepancy between mental self-representation and the actual perception of the audience. The model suggests that because many resources are allocated to detect these negative evaluations, there is less cognitive capacity available to detect or acknowledge positive evaluations from the audience.

Without the InBS-NF paradigm, SAD models consider only the individual response to their own perception of the external cues, e.g., the audience's reaction (Siegel et al., 2018). All the while, individuals that have social anxiety are more likely to perceive neutral, ambiguous, or subtle positive external cues as negative (Morrison and Heimberg, 2013). Whereas, irrefutable and real interpersonal feedback to an individual that act as external cues reduces the risk of the cues being misconceived due to existing social anxiety. Previous research has indicated that there is indeed importance of interpersonal factors that make up a part of social anxiety as a disorder (Siegel et al., 2018). For example, individuals with SAD are empirically more likely to report frequent negative interpersonal events than individuals without (Uliaszek et al., 2010; Farmer and Kashdan, 2012, 2015), demonstrating social anxiety as a predictor of negative interpersonal dependent events (Siegel et al., 2018). These findings stipulate that a relationship exists between interpersonal dependent events and social anxiety. Furthermore, that a gap exists where an interpersonal concept should fill in established SAD models where the inner workings of interpersonal interactions themselves are a contributing factor. Additionally, the outcome of an interpersonal event has been shown to have significant effects on social anxiety, e.g., interpersonal stress (arguments or negative attitudes) is reported to exacerbate and perpetuate symptoms of social anxiety (Alden and Taylor, 2004; Epkins and Heckler, 2011; Fung and Alden, 2017). SAD models outline a cognitive conceptualisation of social anxiety that includes deficiencies in social skills and disproportionate expectations of interactions which lead to anxious anticipation, factors that ultimately play a part in maintaining SAD. These cognitive landmarks facilitate the result of negative interpersonal interactions, subjecting the individual into a vicious cognitive cycle. By this rationale, interpersonal interactions should certainly have a more prominent role in the aetiology and maintenance of SAD, as by doing so clarifies the role of interpersonal measures; and further down the line to help gain insight into the feasibility of 2-PNS to break the cycle of SAD. Whilst the condition of SAD itself is centred around one person, 2-PNS aims to highlight the importance of these individual symptoms being driven by real interpersonal interaction and incorporate the neural activities occurring underneath into models of SAD.

InBS-NF coalesces the dynamics of individual brain activity with an external source that limits the possibility of being subjectively misconstrued by a single individual. That external source being the neural responses from every person in the interaction. For example, an individual with SAD could, by their own pre-dispositions, mistake a neutral response from an audience for being negative (Morrison and Heimberg, 2013). This paradigm allows for a process that can manage symptoms of SAD, by updating their mental self-representation using objective and external cues. InBS-NF facilitates observation and subsequent training and treatment of SAD symptoms in real time, specifically by redirecting the pattern of continued self-deprecation which maintains SAD. The most conventional treatments of SAD that are based on an individual scope include pre-dominantly psychopharmaceuticals (Blanco et al., 2003) and cognitive-behavioural therapy or CBT (Heimberg, 2002). Both avenues have exceptional success within their own right, with up to 77% reported success rates for anti-anxiety medication (Blanco et al., 2013) and 50–70% reported success rate for CBT alone (Clark et al., 2006). Our paradigm largely offers a solution not only as an alternative to the aforementioned treatments, but also to target patients whose circumstances or blockers had placed them in rate of failure. For psychopharmaceutical intervention, an example of a blocker would be the stigma of taking medication to treat a mental illness. Medication for any form of mental illness is still commonly stigmatised as “crazy pills” (Conner and Rosen, 2008) and misrepresented as “zombifying” people by numbing their emotions (Davidson et al., 2008). Whilst inaccurate, patients are still fearful about what perceptions or prejudices others may have of them and often ignore symptoms and treatment in general (Barney et al., 2009). Research has shown an association between self-stigmatism (awareness or agreement with how society stigmatises them) and the discontinuation of medication (Kamaradova et al., 2016). Disempowering the stigma appears to be one of the most important barriers in the beginning a course of psychopharmaceutical treatment (Barney et al., 2009). CBT is an avenue which avoids medication if for those who struggle with overcoming the stigma around medication specifically. The procedure of CBT inherently involves an individual to express in intimate detail their thoughts and feelings. However, there is an oxymoronic trope when an individual with SAD is instructed to open-up to a stranger. The intent and even the initial sessions with a CBT therapist could be just as difficult as the social events that fuel the phobia. For an individual with SAD to successfully open-up in a conversation with a CBT therapist and trusting them could take more effort than other disorders due to the social nature of the SAD (Kaplan et al., 2015). Difficulty to trust and engage with a therapist combined with the persistence of stigma-related thoughts can lead to a higher dropout rate or relapse (Kamaradova et al., 2016). For these patients, InBS-NF can contribute as a placeholder or stepping-stone to in-person therapy. The protocol of InBS-NF is embodied virtually, where research has suggested individuals with SAD can feel more comfortable interacting online or experiencing social situations virtually compared to in-person (High and Caplan, 2009). An individual with SAD has then the opportunity to build trust in the therapist within an initial environment that they are comfortable in; to then increase the chances of success in partaking in CBT by tackling the rudimentary barriers of the specific social situation of opening-up to a therapist. The build-up of trust in a therapist may also contribute to disempowering the stigma for pharmaceutical interventions. Patients are more likely to believe their therapist if the relationship between them is stable (Kaplan et al., 2015), and accept accurate detail and reassurance about medication courses rather than their own assumptions based on society's own stigmatism. Furthermore, InBS-NF protocol provides an individual with an active engagement factor during the simulation where they co-operate with another individual, and have the session monitored by a therapist. In particular, this engagement factor can help reduce feelings of helplessness (Adesola and Li, 2018)—participants are capable of observing changes faster with perceived improvements, and in real-time to maintain and build motivation to continue with the treatment (Ahmed and Westra, 2009). Individuals having an active sense of agency and personal control in a treatment program in addition to seeing results can give power to themselves and combats feelings of helplessness (Soral et al., 2021).

### 4.2. Methods for Interbrain Synchrony and Neurofeedback

**Figure 1:**
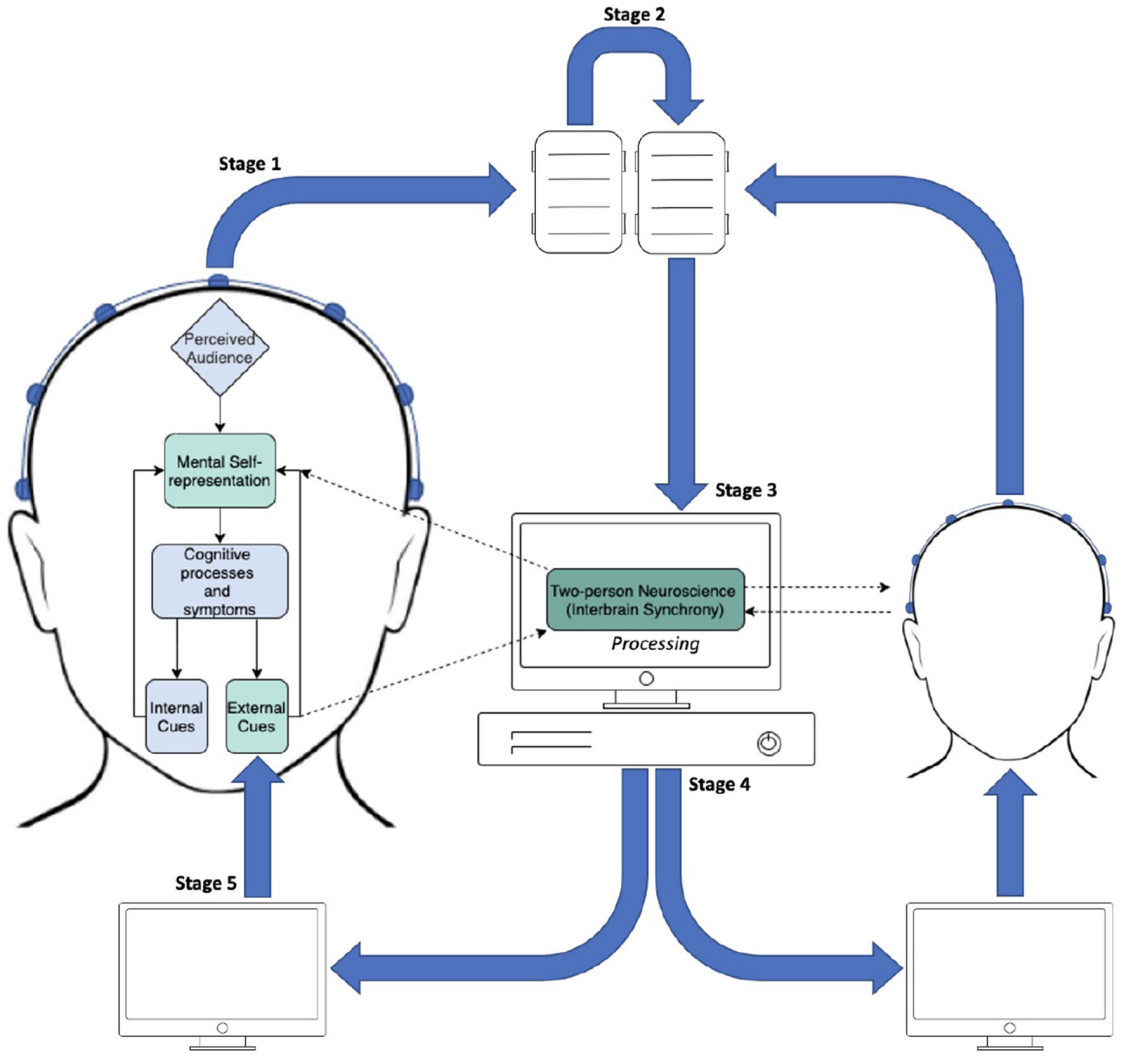
Diagram of the proposed InBS-NF framework. Stage 1: EEG signals being sent from electrodes to amplifiers in two participants separately. Stage 2: daisy-chain link between amplifiers which are connected with different individuals. Stage 3: appended EEG signals from both participants being sent to the processing PC. The text “Processing” refers to a computational framework to calculate the measured degree of phase similarity for InBS. Stage 4: InBS values being sent to interactive simulation (separately for each participant) that represents the InBS values. Stage 5: participants observing InBS values as an external cue.

InBS-NF fundamentally relies on hyperscanning in order to record brain activity on a multi-participant platform (Figure 1). Decades ago, methodological and technological limitations were imposed on hyperscanning techniques and made it difficult to investigate InBS (Babiloni and Astolfi, 2014). In more recent years, it has gradually becoming easier to have reliable resources for hyperscanning in academic labs and technological limitations are less prominent. This became a segue to a surge of InBS studies (including research on SAD) that shared the procedure of calculating synchrony between different brains. Now, limitations are commonly directed towards participant resource or logistics. In section 2.1, research on SAD using different neuroimaging techniques were described. InBS and neurofeedback research have both previously utilised different neuroimaging techniques depending on the task and level of exploration involved. The system framework for InBS-NF proposed in the current review favours particular qualities in neuroimaging due to the nature of real-time feedback. Specifically, InBS-NF requires a naturalistic environment and therefore a high degree of mobility. Additionally, if visual stimuli, through which neurofeedback is provided, constantly change over time responding to InBS or some behavioural measures, the neuroimaging method must be temporally fast and precise enough for feedback signal computation. To meet these requirements, EEG is the most suitable neuroimaging technique. EEG systems are mobile enough to allow naturalistic settings for participants to engage in a social interaction while keeping the recordings resistant to movement artefacts as a result of development of hardware and analysis protocols (Czeszumski et al., 2020).

fMRI scanners falter in performance for InBS-NF in that the set-up has a highly diminished sense of a naturalistic environment and restricted mobility; participants are required to fully lie down in a large and loud scanner, commonly in solitude. Moreover, fMRI analysis suffers from poor temporal resolution which makes precise real-time feedback a strenuous task. In comparison to EEG and fNIRS, fMRI have had less hyperscanning studies due to the fact that it is seldom possible to have multiple fMRI machines available locally to each other (Babiloni and Astolfi, 2014). While fNIRS is indeed a non-invasive technique that offers a high degree of mobility, fNIRS has poor temporal resolution in comparison to EEG and poor spatial resolution in comparison to fMRI. Furthermore, fNIRS (and fMRI) essentially capture metabolic processes (blood oxygen levels), which are only associated with neural activity but not neural activity *per se*. Similarly to EEG, magnetoencephalography (MEG) also measures neural activity (magnetic fields instead of electrical signal as in EEG) and has a high temporal resolution. However, traditional MEG technique uses a large MEG scanner and requires magnetic-field shielding, thus not possible to be mobile and naturalistic. Recent development in the field has seen designs of optically-pumped magnetometer, or OPM-MEG (Boto et al., 2018). OPM-MEG systems are much more mobile and can even be used in virtual reality (e.g., Roberts et al., 2019). However, most OPM-MEG systems available only measure activities from specific brain regions, and more advancement is desired (Hill et al., 2020).

Therefore, EEG is so far the best available technique for the proposed InBS-NF framework, because EEG is capable of measuring neural responses directly with a high temporal resolution in naturalistic settings. This provides us with great timing precision for investigating neural substrates of external stimuli, particularly if experimental stimuli are duration bound. The second best choice is hyperscanning fNIRS on the premise that the protocol pipeline be adjusted to account for the feedback latency (which is significantly longer than that with EEG). For instance, if neurofeedback is only needed for a block of time (e.g., over 10s of seconds). This may be achievable but would be undesirable for many SAD research because social interactions and interaction perceptions may change much more quickly than the fNIRS signal delay in various social situations. Other than EEG and fNIRS, however, current fMRI and MEG (including OPM-MEG) methods would not be suitable in any case for the proposed paradigm.

While the first instance of hyperscanning occurred in the 1960's, EEG hyperscanning has not always been as feasible to carry out as it is in modern day. Recent review papers state that the large influx of multi-participant brain scanning over the past decade are due to technological advances (Nam et al., 2020). Babiloni and Astolfi (2014) state that after the first EEG hyperscanning study, EEG methods largely suffered in spatial sampling and resolution to an extent that it was not conventionally used in research for 40 years. Additionally, a former barrier of EEG included a highly lab-controlled environment necessary for EEG experiments; EEG studies, even on single participants, used to be considered a highly immobile brain imaging method as experimenters had to be very strict on minimising movement of the participant (Babiloni and Astolfi, 2014). However, most EEG hardware manufacturers now offer a mobile alternative which allows for more naturalistic environments (Melnik et al., 2017). Modern recording and signal processing techniques are now sufficient to investigate brain activity accurate to correlate with motor and cognitive behaviour (Michel and Murray, 2012). Algorithms to remove movement artefacts are also available with most EEG softwares, which gives a leniency to movements even for immobile EEG systems. In particular to the social neuroscience field, the theme of research which incorporated EEG studies were predominantly focused on source localisation, determination and the time course of EEG correlates of cognitive processes or behaviour (Koles, 1998). This was tied into the constraints of EEG mobility and capability at the time; it was not always readily available to obtain precisely simultaneous EEG recordings of more than one person. However, off the back of technological advances, there began a new surge in connectomes research (Van Essen et al., 2012). Connectomes represent a comprehensive network of inter-connections across multiple regions in the brain, either anatomically or functionally (Van Essen et al., 2012). Starting with investigations of networks in a single brain, this new direction of research in turn opened up an avenue to investigating functional correlations between networks across brains. EEG is now the most popular method to conduct multi-participant hyperscanning experiments (Czeszumski et al., 2020).

The main limitation of hyperscanning EEG is the low spatial resolution in comparison to fNIRS, and significantly compared to fMRI and MEG. Data obtained can only be inferred (the famous “inverse problem” of EEG localisation) to come from a particular location in the brain (Pascual-Marqui, 1999). The neurofeedback protocol in InBS-NF, i.e., the frequencies and scalp locations to obtain information about brain activity, relies on supporting evidence to justify the relevance of a chosen configuration. That being said, EEG, MEG, fNIRS and fMRI methods have contributed collaboratively in SAD research to help understand the aetiology and maintenance of SAD on a neurological scale (Moscovitch et al., 2011; Tuscan et al., 2013). Each of these methods is capable of extracting different types of information from the brain which link together to provide a full picture. The mechanistic portion of the InBS-NF protocol calls EEG for the most suitable neuroimaging method; however, the training protocol is dependant on a foundation of supporting evidence from previous literature of SAD across all neuroimaging techniques.

**Figure 2:**
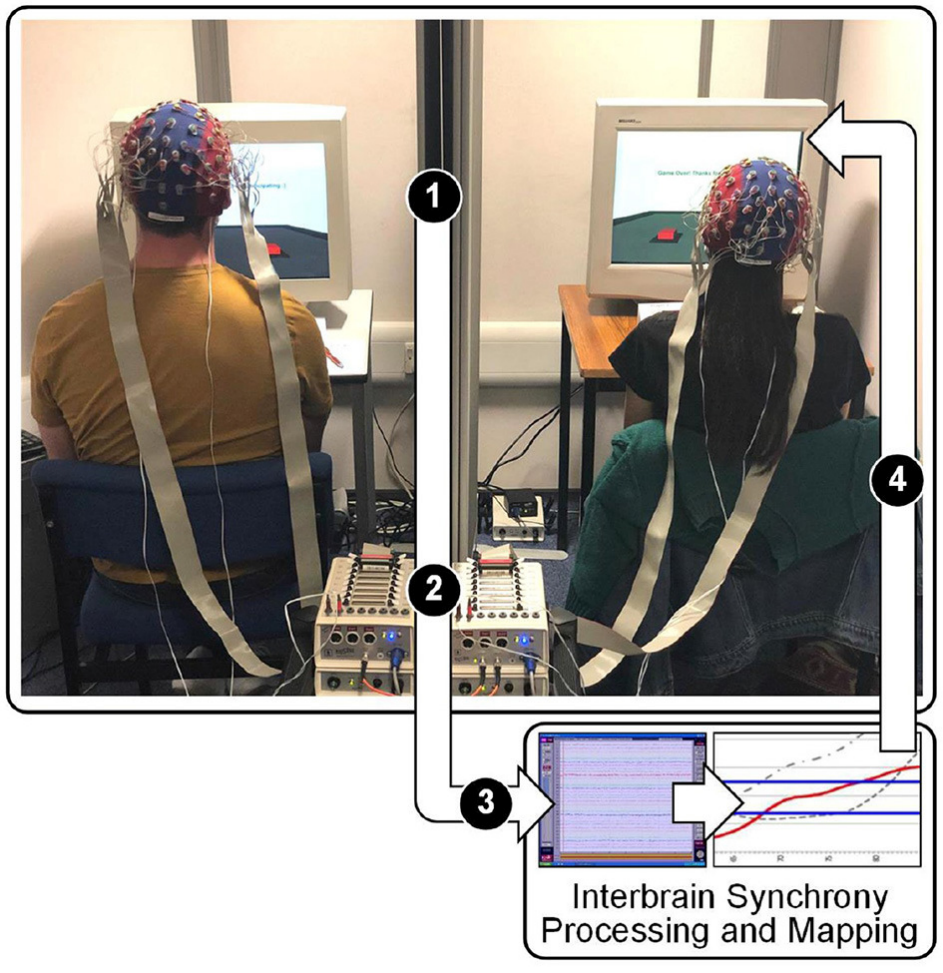
The InBS-NF paradigm: 1) an interactive simulation incorporates the level of interbrain synchronicity shown visually to the participants, 2) raw EEG is measured from both participants is transmitted to the amplifier, 3) InBS is computed from the raw EEG, 4) any changes in synchronicity effects the simulation towards the direction of synchronicity.

The proposed InBS-NF paradigm entails a technique where the degree of synchronicity is fed back via sensory input to each member of a functional unit (dyad) as illustrated in Figure 2. There are three commonly used methods for quantifying neural coupling between brains: the phase-locking value (PLV), the partial directed coherence (PDC) and the imaginary part of coherence (iCoh). For phase-based synchrony the PLV is a mathematical method (Lachaux et al., 1999) for calculating the phase similarity between two or more signals which originate from the same or different electrode sites between the interacting brains. The idea is that the differences between the instantaneous phases of the EEG waveforms involved should remain somewhat constant in synchrony if they are indeed synchronous. The PLV method is widely used in phase-synchrony experiments (Dumas et al., 2010; Yun et al., 2012; Hu et al., 2018). However, non-zero PLVs do not always represent synchrony but rather stem from volume conduction (Li et al., 2020). Volume conduction is the transmission of an electrical potential to multiple locations from a single source throughout a volume conductor (i.e., the brain) (Nunez et al., 1997). The signals spread out over multiple scalp areas are subsequently sampled by EEG devices.

That being said, this issue of volume conduction is not of great concern when PLV is applied to InBS calculation, because the signals cannot spread from one brain to the other in a dyad due to the absence of electric-current conducting connection (such as a cable) between the two participants. If, for some reason, functional connectivity across EEG bands within each single brain is needed for the neurofeedback model—for example, to control for the intrabrain synchrony when analysing InBS—iCoh is recommended (Nolte et al., 2004). iCoh measures coherence to infer synchronicity and by eliminating signals from extraneous sources which stems from instantaneous activity, it is not influenced by volume conduction. What remains from elimination is the true source of interactions, also known as the “imaginary part”. For this reason, this method has gained popularity in recent years (Sander et al., 2010; Sekihara et al., 2011; Doḿınguez et al., 2013). The PDC is also a very commonly used method and was in fact the first approach used in hyperscanning brain-to-brain coupling research (Babiloni et al., 2006). The PDC is particularly useful when determining the direction of synchronicity between interactors in a dyad (Baccalá and Sameshima, 2001) as it computes the direct or indirect causal influences of a signal from one region to a signal in another (Rubinov and Sporns, 2010). The PDC is a frequency-domain measure which analyses interdependences in multivariate networks using autoregressive models (Blinowska et al., 2004; Schelter et al., 2009). Furthermore, the PDC employs a Granger causality statistic (Granger, 1969) which states that if signal *x* influences signal *y*, then the previous values of signal *x* should contain information that is capable of predicting signal *y* more than the previous values of signal *y* itself (Seth, 2005; Seth and Edelman, 2007).

While these quantification methods are capable of deriving a measure of synchronicity between brains with EEG signals, each method measures a different type of synchronicity and can fall into four categories: reciprocal, induced, driven and coincidental (Burgess, 2013). To illustrate, consider there are two signals in which to deduce synchronicity: signal *x* and signal *y*. Reciprocal synchrony refers to the synchrony obtained by a change in signal *x* causing a change in signal *y* and vice versa (thus the influence is bi-directional). Induced synchrony describes an external influence causing synchrony in both signal *x* and signal *y*, such as observing the same scenario in an experiment. Driven synchrony, which is measured by PDC, illustrates that signal *x* influences signal *y* to synchronise. Finally, coincidental synchrony illustrates the synchrony between signal *x* and signal *y* was uninvoked or unintentional. The PLV and iCoh is capable of calculating phase-synchrony; however, no evidence suggests that it can be used to fully distinguish the type of synchronicity which occurs (Burgess, 2013).

### 4.3. Current Standpoint

Brain-to-brain real-time regulation would extend the neurofeedback model to a multi-user platform for training individuals to coordinate, or regulate, their brain activities in line with another person's brain activities. Understanding the effects of this coordination could allow us to understand interpersonal functioning (Gonçalves, 2015) on an implicit scale. Multi-user platforms would benefit the self-regulatory neurofeedback model by means of the social learning models—social interactions, as a learning catalyst, tie into both the social support system (Zhang et al., 2005) and the rewards system (Akers, 2011). Such systems themselves have demonstrated modulation of cortical areas related to social processing (Mathiak et al., 2010). As neurofeedback is inherently a learning process, there is scope for neurofeedback models to improve by introducing interpersonal interaction with a multi-user platform. Even so, self-regulatory neurofeedback maintains a self-centred approach, despite many of our mental states being affected or involve the processes from interpersonal interactions (Gonçalves, 2015).

Currently, there is little evidence illuminating the relationship between InBS and SAD regardless of the potential for understanding the interpersonal aspects of social interaction (Wang et al., 2018). The conceptual frameworks from over a decade-span of InBS studies introduced a potential significance of InBS on behavioural synchronisations and successful communications within the functional unit of interactors (Wang et al., 2018). In particular, the literature provides support for interbrain synchronicity to be very likely reflecting implicit interactions between individuals in a sense of mutual understandings and shared psychological states during a specific social interaction (Cui et al., 2012; Pan et al., 2017). By elucidating the neural correlates of SAD through interpersonal interaction, detection and treatment of the disorder could be improved using the new trajectories of research (Wang et al., 2018) such as localisation of brain regions using a combination of fMRI and EEG methods (Liu et al., 2018). InBS with SAD research should first examine whether the synchrony itself exists within dyads containing one or more individuals with SAD during an interactive task. Secondly, if aberrant synchrony is observed, whether the aberrance can be associated with SAD. And finally, to address a refinement in a neuropsychological model encompassing the interpersonal neural activity for individuals with SAD.

Interbrain interaction, rather than synchrony, has been the main focus of interest to combine with neurofeedback protocols in recent literature. For instance, it was first put forward using an interactive approach, whereby participants would practise motor imagery, recorded by fNIRS, to play a digital game of tug-of-war (Duan et al., 2013) by competitive regulation of certain brain activity. Additionally, portable EEG headsets recording collaborative concentration and collaborative relaxation was achieved using visual feedback on a large-scale experiment in an artistic immersive environment (Kovacevic et al., 2015). The adoption of synchronicity into the interaction platform when the digital tug-of-war game was re-implemented using EEG. The results of the study inferred a relationship between the level of synchronisation of beta-band waves over the central areas of the brain and distinguishing the winner of the game (Zhang and Zhao, 2018). 2-PNS research remains a field which requires further research to establish well-grounded conceptual frameworks and platform validation. The paradigm relies on the premise of both learning through neurofeedback and the understanding of interpersonal interaction through InBS. On that account, both aspects of the technique can be considered as tools. Neurofeedback is the tool used to provide information of the brain activities in real-time, and this information is the tool of synchronicity used as a measure of interpersonal social interaction between the two users.

## 5. Conclusion

In this paper, a prospective 2-PNS approach to SAD is reviewed. SAD was defined using the findings from different neuroimaging techniques (EEG, MEG, fNIRS and fMRI). Topical practises to the treatment of SAD by neurofeedback were described, those being alpha-theta, frontal asymmetry and amygdala modulation. Followed by contemporary InBS interaction frameworks which included joint action, shared attention, affective communication, interactive decision-making and performance indicator.

Our analysis of the current literature strongly suggests that there is a gap in knowledge for both a multi-user neurofeedback platform and an understanding in the role of InBS in SAD. This review supports the importance of the main objective from 2-PNS, that is, identifying an interpersonal aspect of social interactions observed directly from the interactors together compared to individually. These aspects hold the potential to provide valuable insights to solving problems with the treatment of SAD for struggling patients or non-responsive to conventional treatment, and also to other mental disorders with an inherent social deficit quality. As social behaviour evolves in the presence of other people or groups of people (Czeszumski et al., 2020), it is certainly necessary for further research to be conducted that can measure the brain activity in the very moments of social interaction. Thus, successful implementation of the InBS-NF paradigm may have its own standing as a new research model for experimental procedures in 2-PNS.

## Author Contributions

MS, XH, SB, and FC have made substantial, direct, and intellectual contributions to the work and approved for publication. All authors contributed to the article and approved the submitted version.

## Funding

MS was funded by the UK's EPSRC Doctoral Training Programme in Digital Entertainment at the Centre for Digital Entertainment (Bournemouth University) and Applied Neuroscience Solutions Ltd (BrainTrainUK). Grant reference: CDE2: EP/L016540/1.

## Conflict of Interest

SB is the founder and managing director of the company Applied Neuroscience Solutions Ltd. (BrainTrainUK). The remaining authors declare that the research was conducted in the absence of any commercial or financial relationships that could be construed as a potential conflict of interest.

## Publisher's Note

All claims expressed in this article are solely those of the authors and do not necessarily represent those of their affiliated organizations, or those of the publisher, the editors and the reviewers. Any product that may be evaluated in this article, or claim that may be made by its manufacturer, is not guaranteed or endorsed by the publisher.

## References

[B1] Acquadro M. A. Congedo M. De Riddeer D. (2016). Music performance as an experimental approach to hyperscanning studies. Front. Human Neurosci. 10:242. 10.3389/fnhum.2016.00242 27252641 PMC4879135

[B2] Adesola S. A. Li Y. (2018). The relationship between self-regulation, self-efficacy, test anxiety and motivation. Int. J. Inf. Educ. Technol 8, 759–763. 10.18178/ijiet.2018.8.10.1135

[B3] Agha Mohammad Hasani P. Mokhtaree M. Asadollahi Z. Fereidoni M. (2016). The prevalence of social phobia among students of rafsanjan university of medical sciences, iran, and its relation with personality traits in 2013. J. Occupat. Health Epidemiol. 5, 72–82. 10.18869/acadpub.johe.5.2.72

[B4] Ahmed M. Westra H. A. (2009). Impact of a treatment rationale on expectancy and engagement in cognitive behavioral therapy for social anxiety. Cogn. Therapy Res. 33, 314–322. 10.1007/s10608-008-9182-1

[B5] Akers R. L. (2011). Social Learning and Social Structure: A General Theory of Crime and Deviance, 2nd Edn. (New Brunswick, NJ: Transaction Publishers).

[B6] Aladjalova N. A. (1964). Slow electrical processes in the brain, in Progress in Brain Research, Institute of Biophysics, Academy of Sciences, Vol. 7 (Moscow: Elsevier).1204880

[B7] Alden L. E. Taylor C. T. (2004). Interpersonal processes in social phobia. Clin. Psychol. Rev. 24, 857–882. 10.1016/j.cpr.2004.07.006 15501559

[B8] Allen J. J. Coan J. A. Nazarian M. (2004). Issues and assumptions on the road from raw signals to metrics of frontal eeg asymmetry in emotion. Biol. Psychol. 67, 183–218. 10.1016/j.biopsycho.2004.03.007 15130531

[B9] Aranyi G. Cavazza M. Charles F. (2015). Using fnirs for prefrontalasymmetry neurofeedback: methods and challenges, in International Workshop on Symbiotic Interaction (Berlin: Springer), 7–20. 10.1007/978-3-319-24917-92

[B10] Aranyi G. Pecune F. Charles F. Pelachaud C. Cavazza M. (2016). Affective interaction with a virtual character through an fNIRS brain-computer interface. Front. Comput. Neurosci. 10:70. 10.3389/fncom.2016.00070 27462216 PMC4940367

[B11] Arnold P. D. Zai G. Richter M. A. (2004). Genetics of anxiety disorders. Curr. Psychiatry Rep. 6, 243–254. 10.1007/s11920-004-0073-1 15260939

[B12] Astolfi L. Cincotti F. Mattia D. De Vico Fallani F. Salinari S. Marciani M. G.. (2009). Estimation of the cortical activity from simultaneous multi-subject recordings during the prisoner?fs dilemma, in 2009 Annual International Conference of the IEEE Engineering in Medicine and Biology Society (Minneapolis, MA: IEEE), 1937–1939. 10.1109/IEMBS.2009.5333456 19964016

[B13] Astolfi L. Toppi J. Borghini G. Vecchiato G. Isabella R. De Vico Fallani F.. (2011). Study of the functional hyperconnectivity between couples of pilots during flight simulation: An eeg hyperscanning study, in 2011 Annual International Conference of the IEEE Engineering in Medicine and Biology Society (Boston, MA: IEEE), 2338–2341. 10.1109/IEMBS.2011.6090654 22254810

[B14] Astolfi L. Toppi J. Fallani F. D. V. Vecchiato G. Salinari S. Mattia D.. (2010). Neuroelectrical hyperscanning measures simultaneous brain activity in humans. Brain Topography 23, 243–256. 10.1007/s10548-010-0147-9 20480221

[B15] Astolfi L. Toppi J. Vogel P. Mattia D. Babiloni F. Ciaramidaro A.. (2014). Investigating the neural basis of cooperative joint action. an eeg hyperscanning study, in 2014 36th Annual International Conference of the IEEE Engineering in Medicine and Biology Society (Chicago, IL: IEEE), 4896–4899. 10.1109/EMBC.2014.6944721 25571089

[B16] Atzil S. Hendler T. Zagoory-Sharon O. Winetraub Y. Feldman R. (2012). Synchrony and specificity in the maternal and the paternal brain: relations to oxytocin and vasopressin. J. Amer. Acad. Child Adolescent Psychiatry 51, 798–811. 10.1016/j.jaac.2012.06.008 22840551

[B17] Babiloni F. Astolfi L. (2014). Social neuroscience and hyperscanning techniques: past, present and future. Neurosci. Biobehav. Rev. 44, 76–93. 10.1016/j.neubiorev.2012.07.006 22917915 PMC3522775

[B18] Babiloni F. Astolfi L. Cincotti F. Mattia D. Tocci A. Tarantino A.. (2007). Cortical activity and connectivity of human brain during the prisoner?fs dilemma: an eeg hyperscanning study, in 2007 29th Annual International Conference of the IEEE Engineering in Medicine and Biology Society (Lyon: IEEE), 4953–4956. 10.1109/IEMBS.2007.4353452 18003118

[B19] Babiloni F. Cincotti F. Mattia D. Mattiocco M. De Vico Fallani F. Tocci A.. (2006). Hypermethods for eeg hyperscanning, in 2006 International Conference of the IEEE Engineering in Medicine and Biology Society (New York, NY: IEEE), 3666–3669. 10.1109/IEMBS.2006.260754 17945788

[B20] Baccalá L. A. Sameshima K. (2001). Partial directed coherence: a new concept in neural structure determination. Biol. Cybern. 84, 463–474. 10.1007/PL00007990 11417058

[B21] Balconi M. Vanutelli M. E. (2016). Competition in the brain. the contribution of eeg and fnirs modulation and personality effects in social ranking. Front. Psychol. 7:1587. 10.3389/fpsyg.2016.01587 27790181 PMC5062540

[B22] Baldwin D. A. (1995). Understanding the link between joint attention and language, in Joint Attention: Its Origins and Role in Development, eds Moore C. P. J. Dunham, 131–158.

[B23] Barbas H. (2000). Proceedings of the human cerebral cortex: from gene to structure and function connections underlying the synthesis of cognition, memory, and emotion in primate prefrontal cortices. Brain Res 52, 319–330. 10.1016/S0361-9230(99)00245-2 10922509

[B24] Barney L. J. Griffiths K. M. Christensen H. Jorm A. F. (2009). Exploring the nature of stigmatising beliefs about depression and help-seeking: implications for reducing stigma. BMC Public Health 9, 1–11. 10.1186/1471-2458-9-61 19228435 PMC2654888

[B25] Beaton E. A. Schmidt L. A. Ashbaugh A. R. Santesso D. L. Antony M. M. McCabe R. E. (2008). Resting and reactive frontal brain electrical activity (eeg) among a non-clinical sample of socially anxious adults: does concurrent depressive mood matter? Neuropsychiatric Disease Treatment 4, 187. 10.2147/NDT.S1388 18728822 PMC2515916

[B26] Bechara A. Damasio H. Damasio A. R. (2000). Emotion, decision making and the orbitofrontal cortex. Cerebral Cortex 10, 295–307. 10.1093/cercor/10.3.295 10731224

[B27] Beck A. Epstein N. Brown G. Steer R. (1988). An inventory for measuring clinical anxiety: the beck anxiety inventory. J. Consult. Clin. Psychol. 56, 893–897. 10.1037/0022-006X.56.6.893 3204199

[B28] Becker R. Knock S. Ritter P. Jirsa V. (2015). Relating alpha power and phase to population firing and hemodynamic activity using a thalamo-cortical neural mass model. PLoS Comput. Biol. 11:e1004352. 10.1371/journal.pcbi.1004352 26335064 PMC4559309

[B29] Blair K. S. Geraci M. Hollon N. Otero M. DeVido J. Majestic C.. (2010). Social norm processing in adult social phobia: atypically increased ventromedial frontal cortex responsiveness to unintentional (embarrassing) transgressions. Amer. J. Psychiatry 167, 1526–1532. 10.1176/appi.ajp.2010.09121797 20889651 PMC3175630

[B30] Blanco C. Bragdon L. B. Schneier F. R. Liebowitz M. R. (2013). The evidence-based pharmacotherapy of social anxiety disorder. Int. J. Neuropsychopharmacol. 16, 235–249. 10.1017/S1461145712000119 22436306

[B31] Blanco C. Schneier F. R. Schmidt A. Blanco-Jerez C.-R. Marshall R. D. Sánchez-Lacay A.. (2003). Pharmacological treatment of social anxiety disorder: a meta-analysis. Depression Anxiety 18, 29–40. 10.1002/da.10096 12900950

[B32] Blinowska K. J. Kuś R. Kamiński M. (2004). Granger causality and information flow in multivariate processes. Phys. Rev. E 70, 050902. 10.1103/PhysRevE.70.050902 15600583

[B33] Boto E. Holmes N. Leggett J. Roberts G. Shah V. Meyer S. S.. (2018). Moving magnetoencephalography towards real-world applications with a wearable system. Nature 555, 657–661. 10.1038/nature26147 29562238 PMC6063354

[B34] Bressler S. L. Menon V. (2010). Large-scale brain networks in cognition: emerging methods and principles. Trends Cogn. Sci. 14, 277–290. 10.1016/j.tics.2010.04.004 20493761

[B35] Burgess A. P. (2013). On the interpretation of synchronization in eeg hyperscanning studies: a cautionary note. Front. Human Neurosci. 7:881. 10.3389/fnhum.2013.00881 24399948 PMC3870947

[B36] Buxton R. B. Uludağ K. Dubowitz D. J. Liu T. T. (2004). Modeling the hemodynamic response to brain activation. Neuroimage 23, S220–S233. 10.1016/j.neuroimage.2004.07.013 15501093

[B37] Campbell M. J. Schmidt L. A. Santesso D. L. Van Ameringen M. Mancini C. L. Oakman J. M. (2007). Behavioral and psychophysiological characteristics of children of parents with social phobia: a pilot study. Int. J. Neurosci. 117, 605–616. 10.1080/00207450600773780 17464779

[B38] Carpenter M. Nagell K. Tomasello M. Butterworth G. Moore C. (1998). Social cognition, joint attention, and communicative competence from 9 to 15 months of age. Monographs Soc. Res. Child Develop. 63, i–174. 9835078

[B39] Cavazza M. Charles F. Aranyi G. Porteous J. Gilroy S. W. Raz G.. (2014). Towards emotional regulation through neurofeedback, in Proceedings of the 5th Augmented Human International Conference AH '14, Association for Computing Machinery (New York, NY).

[B40] Chapin T. J. Russell-Chapin L. A. (2013). Neurotherapy and Neurofeedback: Brain-based Treatment for Psychological and Behavioral Problems. New York, NY: Routledge. 10.4324/9780203072523

[B41] Chatel-Goldman J. Schwartz J.-L. Jutten C. Congedo M. (2013). Non-local mind from the perspective of social cognition. Front. Human Neurosci. 7:107. 10.3389/fnhum.2013.00107 23565084 PMC3613604

[B42] Clark D. M. Ehlers A. Hackmann A. McManus F. Fennell M. Grey N.. (2006). Cognitive therapy versus exposure and applied relaxation in social phobia: a randomized controlled trial. J. Consult. Clin. Psychol. 74:568. 10.1037/0022-006X.74.3.568 16822113

[B43] Clark D. M. Wells A. (1995). A cognitive model of social phobia, in Social Phobia: Diagnosis, Assessment, and Treatment, eds Heimberg R. G. Liebowitz M. R. Hope D. A. Schneie F. R. (New York, NY: The Guildford Press), 69–93.

[B44] Coan J. A. Allen J. J. (2004). Frontal eeg asymmetry as a moderator and mediator of emotion. Biol. Psychol. 67, 7–50. 10.1016/j.biopsycho.2004.03.002 15130524

[B45] Coan J. A. Schaefer H. S. Davidson R. J. (2006). Lending a hand: social regulation of the neural response to threat. Psychol. Sci. 17, 1032–1039. 10.1111/j.1467-9280.2006.01832.x 17201784

[B46] Cohen A. Keynan J. N. Jackont G. Green N. Rashap I. Shani O.. (2016). Multi-modal virtual scenario enhances neurofeedback learning. Front. Robot. AI 3:52. 10.3389/frobt.2016.00052

[B47] Conner K. O. Rosen D. (2008). you're nothing but a junkie”: multiple experiences of stigma in an aging methadone maintenance population. J. Soc. Work Pract. Addict. 8, 244–264. 10.1080/15332560802157065

[B48] Connor K. M. Davidson J. R. Churchill L. E. Sherwood A. Weisler R. H. Foa E. (2000). Psychometric properties of the social phobia inventory (spin): New self-rating scale. Brit. J. Psychiatry 176, 379–386. 10.1192/bjp.176.4.379 10827888

[B49] Cook I. A. O'Hara R. Uijtdehaage S. H. Mandelkern M. Leuchter A. F. (1998). Assessing the accuracy of topographic eeg mapping for determining local brain function. Electroencephalography Clin. Neurophysiol. 107, 408–414. 10.1016/S0013-4694(98)00092-3 9922086

[B50] Croft R. J. Barry R. J. (2000). Removal of ocular artifact from the eeg: a review. Neurophysiol. Clin. 30, 5–19. 10.1016/S0987-7053(00)00055-1 10740792

[B51] Cui X. Bray S. Bryant D. M. Glover G. H. Reiss A. L. (2011). A quantitative comparison of nirs and fmri across multiple cognitive tasks. Neuroimage 54, 2808–2821. 10.1016/j.neuroimage.2010.10.069 21047559 PMC3021967

[B52] Cui X. Bryant D. M. Reiss A. L. (2012). Nirs-based hyperscanning reveals increased interpersonal coherence in superior frontal cortex during cooperation. Neuroimage 59, 2430–2437. 10.1016/j.neuroimage.2011.09.003 21933717 PMC3254802

[B53] Cunha M. Soares I. Pinto-Gouveia J. (2008). The role of individual temperament, family and peers in social anxiety disorder: a controlled study. Int. J. Clin. Health Psycholo. 8, 631–655.

[B54] Czeszumski A. Eustergerling S. Lang A. Menrath D. Gerstenberger M. Schuberth S.. (2020). Hyperscanning: a valid method to study neural inter-brain underpinnings of social interaction. Front. Hum. Neurosci. 14, 39. 10.3389/fnhum.2020.00039 32180710 PMC7059252

[B55] Dalrymple K. L. (2012). Issues and controversies surrounding the diagnosis and treatment of social anxiety disorder. Exp. Rev. Neurotherapeut. 12, 993–1009. 10.1586/ern.12.81 23002942

[B56] Davidson L. Miller R. Flanagan E. (2008). What's in it for me? the utility of psychiatric treatments from the perspective of the person in recovery. Epidemiol. Psychiatric Sci. 17, 177–181. 10.1017/S1121189X0000124X 18924554

[B57] Davidson R. J. (1992). Anterior cerebral asymmetry and the nature of emotion. Brain Cogn. 20, 125–151. 10.1016/0278-2626(92)90065-T 1389117

[B58] Davidson R. J. (1998). Affective style and affective disorders: perspectives from affective neuroscience. Cogn. Emotion 12, 307–330. 10.1080/026999398379628

[B59] Davidson R. J. (2000). Affective style, psychopathology, and resilience: brain mechanisms and plasticity. Amer. Psychol. 55:1196. 10.1037/0003-066X.55.11.1196 11280935

[B60] Davidson R. J. Ekman P. Saron C. D. Senulis J. A. Friesen W. V. (1990). Approach-withdrawal and cerebral asymmetry: emotional expression and brain physiology: I. J. Personal. Soc. Psychol. 58:330. 10.1037/0022-3514.58.2.330 2319445

[B61] Davidson R. J. Marshall J. R. Tomarken A. J. Henriques J. B. (2000). While a phobic waits: regional brain electrical and autonomic activity in social phobics during anticipation of public speaking. Biol. Psychiatry 47, 85–95. 10.1016/S0006-3223(99)00222-X 10664824

[B62] Dawson G. Toth K. Abbott R. Osterling J. Munson J. Estes A.. (2004). Early social attention impairments in autism: social orienting, joint attention, and attention to distress. Develop. Psychol. 40:271. 10.1037/0012-1649.40.2.271 14979766

[B63] Dikker S. Wan L. Davidesco I. Kaggen L. Oostrik M. McClintock J.. (2017). Brain-to-brain synchrony tracks real-world dynamic group interactions in the classroom. Curr. Biol. 27, 1375–1380. 10.1016/j.cub.2017.04.002 28457867

[B64] Ding J. Chen H. Qiu C. Liao W. Warwick J. M. Duan X.. (2011). Disrupted functional connectivity in social anxiety disorder: a resting-state fmri study. Magn. Resonan. Imag. 29, 701–711. 10.1016/j.mri.2011.02.013 21531100

[B65] Dobrushina O. R. Vlasova R. Pechenkova E.V Rumshiskaya A. D. Litvinova L. D. Mershina E. A.. (2015). The effect of Infra-Low Frequency Neurofeedback on default mode network of the brain” in Conference on Applied Neuroscience and Social Well-Being, Moscow, Russia. p. 27–28. 10.13140/RG.2.1.4272.7122

[B66] Domínguez L. G. Stieben J. Velázquez J. L. P. Shanker S. (2013). The imaginary part of coherency in autism: differences in cortical functional connectivity in preschool children. PLoS One 8:e75941. 10.1371/journal.pone.0075941 24098409 PMC3788049

[B67] Dommer L. Jäger N. Scholkmann F. Wolf M. Holper L. (2012). Between-brain coherence during joint n-back task performance: a two-person functional near-infrared spectroscopy study. Behav. Brain Res. 234, 212–222. 10.1016/j.bbr.2012.06.024 22750679

[B68] Dryman M. T. Gardner S. Weeks J. W. Heimberg R. G. (2016). Social anxiety disorder and quality of life: how fears of negative and positive evaluation relate to specific domains of life satisfaction. J. Anxiety Disorders 38, 1–8. 10.1016/j.janxdis.2015.12.003 26709747

[B69] Duan L. Liu W.-J. Dai R.-N. Li R. Lu C.-M. Huang Y.-X.. (2013). Cross-brain neurofeedback: scientific concept and experimental platform. PLoS One 8:e64590. 10.1371/journal.pone.0064590 23691253 PMC3656856

[B70] Duane T. D. Behrendt T. (1965). Extrasensory electroencephalographic induction between identical twins. Science 150:367. 10.1126/science.150.3694.367 5890891

[B71] Dumas G. Lachat F. Martinerie J. Nadel J. George N. (2011). From social behaviour to brain synchronization: review and perspectives in hyperscanning. Irbm 32, 48–53. 10.1016/j.irbm.2011.01.002

[B72] Dumas G. Nadel J. Soussignan R. Martinerie J. Garnero L. (2010). Inter-brain synchronization during social interaction. PLoS ONE 5:e12166. 10.1371/journal.pone.0012166 20808907 PMC2923151

[B73] Dunbar R. Dunbar R. I. M. Barrett L. (2007). Oxford Handbook of Evolutionary Psychology (New York, NY: Oxford University Press).

[B74] Egner T. Strawson E. Gruzelier J. H. (2002). Eeg signature and phenomenology of alpha/theta neurofeedback training versus mock feedback. Appl. Psychophysiol. Biofeedback 27, 261–270. 10.1023/A:1021063416558 12557453

[B75] Enriquez-Geppert S. Huster R. J. Herrmann C. S. (2017). Eeg-neurofeedback as a tool to modulate cognition and behavior: a review tutorial. Front. Human Neurosci. 11:51. 10.3389/fnhum.2017.00051 28275344 PMC5319996

[B76] Epkins C. C. Heckler D. R. (2011). Integrating etiological models of social anxiety and depression in youth: evidence for a cumulative interpersonal risk model. Clin. Child Family Psychol. Rev. 14, 329–376. 10.1007/s10567-011-0101-8 22080334

[B77] Evans J. R. Abarbanel A. (1999). Introduction to Quantitative Eeg and Neurofeedback (Washington, DC: Elsevier, Academic Press).

[B78] Fallani F. D. V. Nicosia V. Sinatra R. Astolfi L. Cincotti F. Mattia D.. (2010). Defecting or not defecting: how to ‘read' human behavior during cooperative games by eeg measurements. PLoS ONE 5:e14187. 10.1371/journal.pone.0014187 21152069 PMC2995728

[B79] Farmer A. S. Kashdan T. B. (2012). Social anxiety and emotion regulation in daily life: Spillover effects on positive and negative social events. Cogn. Behav. Therapy 41, 152–162. 10.1080/16506073.2012.666561 22428662 PMC3370054

[B80] Farmer A. S. Kashdan T. B. (2015). Stress sensitivity and stress generation in social anxiety disorder: a temporal process approach. J. Abnormal Psychol. 124:102. 10.1037/abn0000036 25688437 PMC4376480

[B81] Funane T. Kiguchi M. Atsumori H. Sato H. Kubota K. Koizumi H. (2011). Synchronous activity of two people's prefrontal cortices during a cooperative task measured by simultaneous near-infrared spectroscopy. J. biomed. Opt. 16:077011. 10.1117/1.3602853 21806291

[B82] Fung K. Alden L. E. (2017). Once hurt, twice shy: social pain contributes to social anxiety. Emotion 17:231. 10.1037/emo0000223 27606825

[B83] García A. M. Ibáñez A. (2014). Two-person neuroscience and naturalistic social communication: the role of language and linguistic variables in brain-coupling research. Front. Psychiatry 5:124. 10.3389/fpsyt.2014.00124 25249986 PMC4155792

[B84] Gentili C. Ricciardi E. Gobbini M. I. Santarelli M. F. Haxby J. V. Pietrini P.. (2009). Beyond amygdala: default mode network activity differs between patients with social phobia and healthy controls. Brain Res. Bull. 79, 409–413. 10.1016/j.brainresbull.2009.02.002 19559343

[B85] George L. Lecuyer A. (2010). An overview of research on passive brain-computer interfaces for implicit human-computer interaction, in International Conference on Applied Bionics and Biomechanics ICABB 2010-Workshop W1 Brain-Computer Interfacing and Virtual Reality (Venice: ICABB).

[B86] Glassman L. H. Kuster A. T. Shaw J. A. Forman E. M. Izzetoglu M. Matteucci A.. (2017). The relationship between dorsolateral prefrontal activation and speech performance-based social anxiety using functional near infrared spectroscopy. Brain Imag. Behav. 11, 797–807. 10.1007/s11682-016-9554-1 27180247

[B87] Goldway N. Ablin J. Lubin O. Zamir Y. Keynan J. N. Or-Borichev A.. (2019). Volitional limbic neuromodulation exerts a beneficial clinical effect on fibromyalgia. NeuroImage 186, 758–770. 10.1016/j.neuroimage.2018.11.001 30408596

[B88] Gonçalves O. F. (2015). The emergence of interpersonal neuro modulation: from single to mutual brainregulation, in Counselling Today, Vol. 58 (New York, NY: Wiley), 16–21.

[B89] Granger C. W. (1969). Some recent developments in a concept of causality. J. Econometr. 39, 199–211.

[B90] Grecucci A. Giorgetta C. Brambilla P. Zuanon S. Perini L. Balestrieri M.. (2013). Anxious ultimatums: how anxiety disorders affect socioeconomic behaviour. Cogn. Emotion 27, 230–244. 10.1080/02699931.2012.698982 22775394

[B91] Grin-Yatsenko V. Ponomarev V. Kara O. Wandernoth B. Gregory M. Ilyukhina V.. (2018b). Effect of infra-low frequency neurofeedback on infra-slow eeg fluctuations (St Petersburg: Intechopen). 10.5772/intechopen.77154

[B92] Grin-Yatsenko V. A. Othmer S. Ponomarev V. A. Evdokimov S. A. Konoplev Y. Y. Kropotov J. D. (2018a). Infra-low frequency neurofeedback in depression: Three case studies. NeuroRegulation 5, 30–30. 10.15540/nr.5.1.30

[B93] Gruzelier J. (2009). A theory of alpha/theta neurofeedback, creative performance enhancement, long distance functional connectivity and psychological integration. Cogn. Process. 10, 101–109. 10.1007/s10339-008-0248-5 19082646

[B94] Gutz L. Roepke S. Renneberg B. (2016). Cognitive and affective processing of social exclusion in borderline personality disorder and social anxiety disorder. Behav. Res. Therapy 87, 70–75. 10.1016/j.brat.2016.08.020 27616717

[B95] Guyer A. E. Lau J. Y. McClure-Tone E. B. Parrish J. Shiffrin N. D. Reynolds R. C.. (2008). Amygdala and ventrolateral prefrontal cortex function during anticipated peer evaluation in pediatric social anxiety. Arch. Gen. Psychiatry 65, 1303–1312. 10.1001/archpsyc.65.11.1303 18981342 PMC2717208

[B96] Gwin J. T. Gramann K. Makeig S. Ferris D. P. (2010). Removal of movement artifact from high-density eeg recorded during walking and running. J. Neurophysiol. 103, 3526–3534. 10.1152/jn.00105.2010 20410364 PMC3774587

[B97] Hahn A. Stein P. Windischberger C. Weissenbacher A. Spindelegger C. Moser E.. (2011). Reduced resting-state functional connectivity between amygdala and orbitofrontal cortex in social anxiety disorder. Neuroimage 56, 881–889. 10.1016/j.neuroimage.2011.02.064 21356318

[B98] Hammond D. C. (2007). What is neurofeedback? J. Neurotherapy 10, 25–36. 10.1300/J184v10n04_04

[B99] Hampel S. Weis S. Hiller W. Witthöft M. (2011). The relations between social anxiety and social intelligence: A latent variable analysis. J. Anxiety Disorders 25, 545–553. 10.1016/j.janxdis.2011.01.001 21315550

[B100] Hannesdóttir D. K. Doxie J. Bell M. A. Ollendick T. H. Wolfe C. D. (2010). A longitudinal study of emotion regulation and anxiety in middle childhood: associations with frontal eeg asymmetry in early childhood. Dev. Psychobiol. J. Int. Soc. Dev. Psychobiol. 52, 197–204. 10.1002/dev.20425 20112261

[B101] Hari R. Kujala M. V. (2009). Brain basis of human social interaction: from concepts to brain imaging. Physiol. Rev. 89, 453–479. 10.1152/physrev.00041.2007 19342612

[B102] Harrewijn A. Van der Molen M. Westenberg P. (2016). Putative eeg measures of social anxiety: comparing frontal alpha asymmetry and delta–beta cross-frequency correlation. Cogn. Affect. Behav. Neurosci. 16, 1086–1098. 10.3758/s13415-016-0455-y 27557885 PMC5153416

[B103] Hasan J. Broughton R. (1994). Quantitative topographic eeg mapping during drowsiness and sleep onset, in Sleep Onset: Normal and Abnormal Processes, eds Ogilvie R. D. Harsh J. R. (New York, NY: American Psychological Association), 219–235. 10.1037/10166-013

[B104] Hasson U. Ghazanfar A. A. Galantucci B. Garrod S. Keysers C. (2012). Brain-to-brain coupling: a mechanism for creating and sharing a social world. Trends Cogn. Sci. 16, 114–121. 10.1016/j.tics.2011.12.007 22221820 PMC3269540

[B105] Heimberg R. G. (2002). Cognitive-behavioral therapy for social anxiety disorder: current status and future directions. Biol. Psychiatry 51, 101–108. 10.1016/S0006-3223(01)01183-0 11801235

[B106] Herrmann C. S. Strüber D. Helfrich R. F. Engel A. K. (2016). Eeg oscillations: from correlation to causality. Int. J. Psychophysiol. 103, 12–21. 10.1016/j.ijpsycho.2015.02.003 25659527

[B107] Herwig U. Lutz J. Scherpiet S. Scheerer H. Kohlberg J. Opialla S.. (2019). Training emotion regulation through real-time fmri neurofeedback of amygdala activity. NeuroImage 184, 687–696. 10.1016/j.neuroimage.2018.09.068 30287300

[B108] High A. C. Caplan S. E. (2009). Social anxiety and computer-mediated communication during initial interactions: implications for the hyperpersonal perspective. Comput. Human Behav. 25, 475–482. 10.1016/j.chb.2008.10.011

[B109] Hill R. M. Boto E. Rea M. Holmes N. Leggett J. Coles L. A.. (2020). Multi-channel whole-head opm-meg: helmet design and a comparison with a conventional system. NeuroImage 219:116995. 10.1016/j.neuroimage.2020.116995 32480036 PMC8274815

[B110] Hiltunen T. Kantola J. Abou Elseoud A. Lepola P. Suominen K. Starck T.. (2014). Infra-slow eeg fluctuations are correlated with resting-state network dynamics in fmri. J. Neurosci. 34, 356–362. 10.1523/JNEUROSCI.0276-13.2014 24403137 PMC6608153

[B111] Hjorth B. (1970). Eeg analysis based on time domain properties. Electroencephalography Clin. Neurophysiol. 29, 306–310. 10.1016/0013-4694(70)90143-4 4195653

[B112] Hobson H. M. Bishop D. V. (2017). The interpretation of mu suppression as an index of mirror neuron activity: past, present and future. Roy. Soc. Open Sci. 4:160662. 10.1098/rsos.160662 28405354 PMC5383811

[B113] Hu Y. Hu Y. Li X. Pan Y. Cheng X. (2017). Brain-to-brain synchronization across two persons predicts mutual prosociality. Soc. Cogn. Affect. Neurosci. 12, 1835–1844. 10.1093/scan/nsx118 29040766 PMC5716073

[B114] Hu Y. Pan Y. Shi X. Cai Q. Li X. Cheng X. (2018). Inter-brain synchrony and cooperation context in interactive decision making. Biol. Psychol. 133, 54–62. 10.1016/j.biopsycho.2017.12.005 29292232

[B115] Jahng J. Kralik J. D. Hwang D.-U. Jeong J. (2017). Neural dynamics of two players when using nonverbal cues to gauge intentions to cooperate during the prisoner's dilemma game. Neuroimage 157, 263–274. 10.1016/j.neuroimage.2017.06.024 28610901

[B116] Jarallah H. Al-Omari F. Altowairiqi I. Al Saadi K. (2017). Magnitude of social anxiety disorder, and impact on quality of life among medical students, taif city-ksa. J. Psychol. Clin. Psychiatry 7:00454. 10.15406/jpcpy.2017.07.00454

[B117] Jeunet C. Debener S. Lotte F. Mattout J. Scherer R. Zich C. (2018). Mind the traps! design guidelines for rigorous bci experiments, in Braincomputer Interfaces Handbook: Technological and Theoretical Advances, eds Nam C. S. Nijholt A. Lotte F. (Cham: CRC Press), 613–618. Available Online at: https://www.taylorfrancis.com/books/e/9781351231947/chapters/10.1201%2F9781351231954-45

[B118] Jiang J. Chen C. Dai B. Shi G. Ding G. Liu L.. (2015). Leader emergence through interpersonal neural synchronization. Proc. Natl. Acad. Sci. U.S.A. 112, 4274–4279. 10.1073/pnas.1422930112 25831535 PMC4394311

[B119] Jiang J. Dai B. Peng D. Zhu C. Liu L. Lu C. (2012). Neural synchronization during face-to-face communication. J. Neurosci. 32, 16064–16069. 10.1523/JNEUROSCI.2926-12.2012 23136442 PMC6621612

[B120] Jirsa V. Müller V. (2013). Cross-frequency coupling in real and virtual brain networks. Front. Comput. Neurosci. 7:78. 10.3389/fncom.2013.00078 23840188 PMC3699761

[B121] Johnson H. D. Lavoie J. C. Mahoney M. (2001). Interparental conflict and family cohesion: Predictors of loneliness, social anxiety, and social avoidance in late adolescence. J. Adolescent Res. 16, 304–318. 10.1177/0743558401163004

[B122] Kaiboriboon K. Lüders H. O. Hamaneh M. Turnbull J. Lhatoo S. D. (2012). Eeg source imaging in epilepsy practicalities and pitfalls. Nat. Rev. Neurol. 8, 498–507. 10.1038/nrneurol.2012.150 22868868

[B123] Kaiser D. A. (2005). Basic principles of quantitative eeg. J. Adult Dev. 12, 99–104. 10.1007/s10804-005-7025-9

[B124] Kamaradova D. Latalova K. Prasko J. Kubinek R. Vrbova K. Mainerova B.. (2016). Connection between self-stigma, adherence to treatment, and discontinuation of medication. Patient Preference Adherence 10:1289. 10.2147/PPA.S99136 27524884 PMC4966500

[B125] Kaplan S. C. Levinson C. A. Rodebaugh T. L. Menatti A. Weeks J. W. (2015). Social anxiety and the big five personality traits: the interactive relationship of trust and openness. Cogn. Behav. Therapy 44, 212–222. 10.1080/16506073.2015.1008032 25705989

[B126] Kawano T. Ishigame A. Majima Y. Maekawa Y. Katagiri M. (2016). Inter-brain synchronization between nurse and patient during drawing blood, in Proceedings of the International Joint Conference on Biomedical Engineering Systems and Technologies (BIOSTEC 2016) (Setubal: SCITEPRESS - Science and Technology Publications), 507–511. 10.5220/0005825605070511

[B127] Kawasaki M. Yamada Y. Ushiku Y. Miyauchi E. Yamaguchi Y. (2013). Inter-brain synchronization during coordination of speech rhythm in human-to-human social interaction. Sci. Rep. 3, 1–8. 10.1038/srep01692 23603749 PMC3631767

[B128] Kent G. Keohane S. (2001). Social anxiety and disfigurement: the moderating effects of fear of negative evaluation and past experience. Brit. J. Clin. Psychol. 40, 23–34. 10.1348/014466501163454 11317946

[B129] Kerson C. Sherman R. A. Kozlowski G. P. (2009). Alpha suppression and symmetry training for generalized anxiety symptoms. J. Neurotherapy 13, 146–155. 10.1080/10874200903107405

[B130] Kessler R. C. Stein M. B. Berglund P. (1998). Social phobia subtypes in the national comorbidity survey. Amer. J. Psychiatry 155, 613–619. 10.1176/ajp.155.5.613 9585711

[B131] Kim M. J. Whalen P. J. (2009). The structural integrity of an amygdala–prefrontal pathway predicts trait anxiety. J. Neurosci. 29, 11614–11618. 10.1523/JNEUROSCI.2335-09.2009 19759308 PMC2791525

[B132] Kim Y.-K. Yoon H.-K. (2018). Common and distinct brain networks underlying panic and social anxiety disorders. Progress Neuro Psychopharmacol. Biol. Psychiatry 80, 115–122. 10.1016/j.pnpbp.2017.06.017 28642079

[B133] Kinreich S. Djalovski A. Kraus L. Louzoun Y. Feldman R. (2017). Brain-to-brain synchrony during naturalistic social interactions. Sci. Rep. 7, 1–12. 10.1038/s41598-017-17339-5 29213107 PMC5719019

[B134] Kleberg J. L. Högström J. Nord M. Bölte S. Serlachius E. Falck-Ytter T. (2017). Autistic traits and symptoms of social anxiety are differentially related to attention to others eyes in social anxiety disorder. J. Autism Dev. Disord. 47, 3814–3821. 10.1007/s10803-016-2978-z 28000078 PMC5676829

[B135] Klumpp H. Angstadt M. Nathan P. J. Phan K. L. (2010). Amygdala reactivity to faces at varying intensities of threat in generalized social phobia: an event-related functional mri study. Psychiatry Res. Neuroimaging 183, 167–169. 10.1016/j.pscychresns.2010.05.001 20609570 PMC2911140

[B136] Klumpp H. Angstadt M. Phan K. L. (2012). Insula reactivity and connectivity to anterior cingulate cortex when processing threat in generalized social anxiety disorder. Biol. Psychol. 89, 273–276. 10.1016/j.biopsycho.2011.10.010 22027088 PMC3260042

[B137] Klumpp H. Fitzgerald D. A. Phan K. L. (2013). Neural predictors and mechanisms of cognitive behavioral therapy on threat processing in social anxiety disorder. Progress Neuro Psychopharmacol. Biol. Psychiatry 45, 83–91. 10.1016/j.pnpbp.2013.05.004 23665375 PMC3951971

[B138] Knoblich G. Butterfill S. Sebanz N. (2011). Psychological research on joint action: theory and data. Psycho. Learn. Motivat. 54, 59–101. 10.1016/B978-0-12-385527-5.00003-6

[B139] Koike T. Tanabe H. C. Sadato N. (2015). Hyperscanning neuroimaging technique to reveal the two-in-one system in social interactions. Neurosci. Res. 90, 25–32. 10.1016/j.neures.2014.11.006 25499683

[B140] Koles Z. J. (1998). Trends in eeg source localization. Electroencephal. Clin. Neurophysiol. 106, 127–137. 10.1016/S0013-4694(97)00115-6 9741773

[B141] Konvalinka I. Bauer M. Stahlhut C. Hansen L. K. Roepstorff A. Frith C. D. (2014). Frontal alpha oscillations distinguish leaders from followers: multivariate decoding of mutually interacting brains. Neuroimage 94, 79–88. 10.1016/j.neuroimage.2014.03.003 24631790

[B142] Konvalinka I. Roepstorff A. (2012). The two-brain approach: how can mutually interacting brains teach us something about social interaction? Front. Human Neurosci. 6:215. 10.3389/fnhum.2012.00215 22837744 PMC3402900

[B143] Kovacevic N. Ritter P. Tays W. Moreno S. McIntosh A. R. (2015). ‘my virtual dream': collective neurofeedback in an immersive art environment. PLoS ONE 10:e0130129. 10.1371/journal.pone.0130129 26154513 PMC4496007

[B144] Krill A. L. Platek S. M. (2012). Working together may be better: activation of reward centers during a cooperative maze task. PLoS ONE 7:e30613. 10.1371/journal.pone.0030613 22355319 PMC3280262

[B145] Kropotov J. D. (2010). Quantitative eeg, event-related potentials and neurotherapy (St Petersburg), 1–505. 10.1016/B978-0-12-374512-5.X0001-126706985

[B146] Kropotov J. D. (2016). Functional neuromarkers for psychiatry: Applications for diagnosis and treatment, in Functional Neuromarkers for Psychiatry: Applications for Diagnosis and Treatment (St. Petersburg), 1–462. 10.1016/C2012-0-07144-X

[B147] Kuo J. R. Goldin P. R. Werner K. Heimberg R. G. Gross J. J. (2011). Childhood trauma and current psychological functioning in adults with social anxiety disorder. J. Anxiety Disord. 25, 467–473. 10.1016/j.janxdis.2010.11.011 21183310 PMC3074005

[B148] Lőrincz M. L. Geall F. Bao Y. Crunelli V. Hughes S. W. (2009). Atp-dependent infra-slow (< 0.1 hz) oscillations in thalamic networks. PLoS ONE 4:e4447. 10.1371/journal.pone.0004447 19212445 PMC2637539

[B149] Lachaux J.-P. Rodriguez E. Martinerie J. Varela F. J. (1999). Measuring phase synchrony in brain signals. Human Brain Mapp. 8, 194–208. 10.1002/(sici)1097-0193(1999)8:4<194::aid-hbm4>3.0.co;2-c 10619414 PMC6873296

[B150] Lahnakoski J. M. Glerean E. Jääskeläinen I. P. Hyönä J. Hari R. Sams M.. (2014). Synchronous brain activity across individuals underlies shared psychological perspectives. Neuroimage 100, 316–324. 10.1016/j.neuroimage.2014.06.022 24936687 PMC4153812

[B151] Legarda S. B. McMahon D. Othmer S. Othmer S. (2011). Clinical neurofeedback: case studies, proposed mechanism, and implications for pediatric neurology practice. J. Child Neurol. 26, 1045–1051. 10.1177/0883073811405052 21576401

[B152] Li X. Wu Y. Wei M. Guo Y. Yu Z. Wang H.. (2020). A novel index of functional connectivity: phase lag based on wilcoxon signed rank test. Cogn. Neurodyn. 15, 621–636. 10.1007/s11571-020-09646-x 34367364 PMC8286916

[B153] Li Z. Tong L. Guan M. He W. Wang L. Bu H.. (2016). Altered resting-state amygdala functional connectivity after real-time fmri emotion self-regulation training. BioMed Res. Int. 2016:2719895. 10.1155/2016/2719895 26998482 PMC4779507

[B154] Liao W. Qiu C. Gentili C. Walter M. Pan Z. Ding J.. (2010). Altered effective connectivity network of the amygdala in social anxiety disorder: a resting-state fmri study. PloS one 5:e15238. 10.1371/journal.pone.0015238 21203551 PMC3008679

[B155] Liebowitz M. R. Heimberg R. G. Schneier F. R. Hope D. A. Davies S. Holt C. S.. (1999). Cognitive-behavioral group therapy versus phenelzine in social phobia: long term outcome. Depress. Anxiety 10, 89–98. 10.1002/(SICI)1520-6394(1999)10:3<89::AID-DA1>3.0.CO;2-5 10604081

[B156] Lindenberger U. Li S.-C. Gruber W. Müller V. (2009). Brains swinging in concert: cortical phase synchronization while playing guitar. BMC Neurosci. 10, 1–12. 10.1186/1471-2202-10-22 19292892 PMC2662862

[B157] Liu D. Liu S. Liu X. Zhang C. Li A. Jin C.. (2018). Interactive brain activity: review and progress on eeg-based hyperscanning in social interactions. Front. Psychol. 9:1862. 10.3389/fpsyg.2018.01862 30349495 PMC6186988

[B158] Maner J. K. Schmidt N. B. (2006). The role of risk avoidance in anxiety. Behav. Therapy 37, 181–189. 10.1016/j.beth.2005.11.003 16942970

[B159] Marks I. M. Gelder M. (1966). Different ages of onset in varieties of phobia. Amer. J. Psychiatry 123, 218–221. 10.1176/ajp.123.2.218 5944004

[B160] Marzbani H. Marateb H. R. Mansourian M. (2016). Neurofeedback: a comprehensive review on system design, methodology and clinical applications. Basic Clin. Neurosci. 7:143. 10.15412/J.BCN.03070208 27303609 PMC4892319

[B161] Mathiak K. A. Koush Y. Dyck M. Gaber T. J. Alawi E. Zepf F. D.. (2010). Social reinforcement can regulate localized brain activity. Eur. Archives Psychiatry Clin. Neurosci. 260, 132–136. 10.1007/s00406-010-0135-9 20936298

[B162] McAssey M. P. Helm J. Hsieh F. Sbarra D. A. Ferrer E. (2013). Methodological advances for detecting physiological synchrony during dyadic interactions. Methodol. Eur. J. Res. Methods Behav. Soc. Sci. 9:41. 10.1027/1614-2241/a000053

[B163] Meier S. M. Mattheisen M. Mors O. Mortensen P. B. Laursen T. M. Penninx B. W. (2016). Increased mortality among people with anxiety disorders: total population study. The Brit. J. Psychiatry 209, 216–221. 10.1192/bjp.bp.115.171975 27388572 PMC5082973

[B164] Meir-Hasson Y. Kinreich S. Podlipsky I. Hendler T. Intrator N. (2014). An eeg finger-print of fmri deep regional activation. Neuroimage 102, 128–141. 10.1016/j.neuroimage.2013.11.004 24246494

[B165] Melnik A. Legkov P. Izdebski K. Kärcher S. M. Hairston W. D. Ferris D. P.. (2017). Systems, subjects, sessions: to what extent do these factors influence eeg data? Front. Human Neurosci. 11:150. 10.3389/fnhum.2017.00150 28424600 PMC5371608

[B166] Mennella R. Patron E. Palomba D. (2017). Frontal alpha asymmetry neurofeedback for the reduction of negative affect and anxiety. Behav. Res. Therapy 92, 32–40. 10.1016/j.brat.2017.02.002 28236680

[B167] Menon V. (2011). Large-scale brain networks and psychopathology: a unifying triple network model. Trends Cogn. Sci. 15, 483–506. 10.1016/j.tics.2011.08.003 21908230

[B168] Ménoret M. Varnet L. Fargier R. Cheylus A. Curie A. Des Portes V.. (2014). Neural correlates of non-verbal social interactions: a dual-eeg study. Neuropsychologia 55, 85–97. 10.1016/j.neuropsychologia.2013.10.001 24157538

[B169] Michel C. M. Murray M. M. (2012). Towards the utilization of eeg as a brain imaging tool. Neuroimage 61, 371–385. 10.1016/j.neuroimage.2011.12.039 22227136

[B170] Millan J. D. R. Galan F. Vanhooydonck D. Lew E. Philips J. Nuttin M. (2009). Asynchronous non-invasive brain-actuated control of an intelligent wheelchair, in 2009 Annual International Conference of the IEEE Engineering in Medicine and Biology Society (Minneapolis, MA: IEEE), 3361–3364. 10.1109/IEMBS.2009.5332828 19963794

[B171] Miskovic V. Ashbaugh A. R. Santesso D. L. McCabe R. E. Antony M. M. Schmidt L. A. (2010). Frontal brain oscillations and social anxiety: A cross-frequency spectral analysis during baseline and speech anticipation. Biol. Psychol. 83, 125–132. 10.1016/j.biopsycho.2009.11.010 19945500

[B172] Miskovic V. Campbell M. J. Santesso D. L. Van Ameringen M. Mancini C. L. Schmidt L. A. (2011a). Frontal brain oscillatory coupling in children of parents with social phobia: a pilot study. J. Neuropsychiatry Clin. Neurosci. 23, 111–114. 10.1176/appi.neuropsych.23.1.111 21304147

[B173] Miskovic V. Moscovitch D. A. Santesso D. L. McCabe R. E. Antony M. M. Schmidt L. A. (2011b). Changes in eeg cross-frequency coupling during cognitive behavioral therapy for social anxiety disorder. Psychol. Sci. 22, 507–516. 10.1177/0956797611400914 21378369

[B174] Montague P. R. Berns G. S. Cohen J. D. McClure S. M. Pagnoni G. Dhamala M.. (2002). Hyperscanning: simultaneous fmri during linked social interactions. Neuroimage 16, 1159–1164. 12202103 10.1006/nimg.2002.1150

[B175] Morrison A. S. Heimberg R. G. (2013). Social anxiety and social anxiety disorder. Ann. Rev. Clin. Psychol. 9, 249–274. 10.1146/annurev-clinpsy-050212-185631 23537485

[B176] Moscovitch D. A. Santesso D. L. Miskovic V. McCabe R. E. Antony M. M. Schmidt L. A. (2011). Frontal eeg asymmetry and symptom response to cognitive behavioral therapy in patients with social anxiety disorder. Biol. Psychol. 87, 379–385. 10.1016/j.biopsycho.2011.04.009 21571033

[B177] Mundy P. Acra C. F. (2006). Joint attention, social engagement, and the development of social competence, in The Development of Social Engagement: Neurobiological Perspectives, 81–117.

[B178] Mundy P. Gomes A. (1998). Individual differences in joint attention skill development in the second year. Infant Behav. Develop. 21, 469–482. 10.1016/S0163-6383(98)90020-0

[B179] Mundy P. Newell L. (2007). Attention, joint attention, and social cognition. Curr. Direct. Psychol. Sci. 16, 269–274. 10.1111/j.1467-8721.2007.00518.x 19343102 PMC2663908

[B180] Mundy P. Sigman M. (2006). Joint attention, social competence, and developmental psychopathology, in Developmental Psychopathology: Theory and Method, eds Cicchetti D. Cohen D. J. (John Wiley & Sons, Inc.), 293–332.

[B181] Muthukumaraswamy S. (2013). High-frequency brain activity and muscle artifacts in meg/eeg: a review and recommendations. Front. Human Neurosci. 7:138. 10.3389/fnhum.2013.00138 23596409 PMC3625857

[B182] Nakao T. Sanematsu H. Yoshiura T. Togao O. Murayama K. Tomita M.. (2011). fmri of patients with social anxiety disorder during a social situation task. Neurosci. Res. 69, 67–72. 10.1016/j.neures.2010.09.008 20888872

[B183] Nam C. S. Choo S. Huang J. Park J. (2020). Brain-to-brain neural synchrony during social interactions: a systematic review on hyperscanning studies. Appl. Sci. 10:6669. 10.3390/app10196669

[B184] Nolte G. Bai O. Wheaton L. Mari Z. Vorbach S. Hallett M. (2004). Identifying true brain interaction from eeg data using the imaginary part of coherency. Clin. Neurophysiol. 115, 2292–2307. 10.1016/j.clinph.2004.04.029 15351371

[B185] Norton A. R. Abbott M. J. (2016). Self-focused cognition in social anxiety: a review of the theoretical and empirical literature. Behav. Change 33, 44–64. 10.1017/bec.2016.230886898

[B186] Nunez P. L. Srinivasan R. Westdorp A. F. Wijesinghe R. S. Tucker D. M. Silberstein R. B.. (1997). Eeg coherency: I: statistics, reference electrode, volume conduction, laplacians, cortical imaging, and interpretation at multiple scales. Electroencephalography Clin. Neurophysiol. 103, 499–515. 10.1016/S0013-4694(97)00066-7 9402881

[B187] Oakes T. R. Pizzagalli D. A. Hendrick A. M. Horras K. A. Larson C. L. Abercrombie H. C.. (2004). Functional coupling of simultaneous electrical and metabolic activity in the human brain. Human Brain Mapp. 21, 257–270. 10.1002/hbm.20004 15038007 PMC6871925

[B188] Ochsner K. N. (2004). Current directions in social cognitive neuroscience. Curr. Opin. Neurobiol. 14, 254–258. 10.1016/j.conb.2004.03.011 15082333

[B189] Osaka N. Minamoto T. Yaoi K. Azuma M. Shimada Y. M. Osaka M. (2015). How two brains make one synchronized mind in the inferior frontal cortex: fnirs-based hyperscanning during cooperative singing. Front. Psychol. 6:1811. 10.3389/fpsyg.2015.01811 26635703 PMC4659897

[B190] Othmer S. Othmer S. (2016). Infra-low-frequency neurofeedback for optimum performance. Biofeedback 44, 81–89. 10.5298/1081-5937-44.2.07

[B191] Othmer S. Othmer S. F. Kaiser D. A. Putman J. (2013). Endogenous neuro modulation at infralow frequencies. Semin. Pediatr. Neurol. 20, 246–257. 10.1016/j.spen.2013.10.006 24365573

[B192] Palva J. M. Palva S. (2012). Infra-slow fluctuations in electrophysiological recordings, blood-oxygenation-level-dependent signals, and psychophysical time series. Neuroimage 62, 2201–2211. 10.1016/j.neuroimage.2012.02.060 22401756

[B193] Pan Y. Cheng X. Zhang Z. Li X. Hu Y. (2017). Cooperation in lovers: an f nirs-based hyperscanning study. Human Brain Mapping 38, 831–841. 10.1002/hbm.23421 27699945 PMC6867051

[B194] Parlade M. V. Messinger D. S. Delgado C. E. Kaiser M. Y. Van Hecke A. V. Mundy P. C. (2009). Anticipatory smiling: Linking early affective communication and social outcome. Infant Behav. Develop. 32, 33–43. 10.1016/j.infbeh.2008.09.007 19004500 PMC2650826

[B195] Parri H. R. Crunelli V. (2001). Pacemaker calcium oscillations in thalamic astrocytes in situ. Neuroreport 12, 3897–3900. 10.1097/00001756-200112210-00008 11742206

[B196] Parri H. R. Gould T. M. Crunelli V. (2001). Spontaneous astrocytic ca 2+ oscillations in situ drive nmdar-mediated neuronal excitation. Nat. Neurosci. 4, 803–812. 10.1038/90507 11477426

[B197] Pascual-Marqui R. D. (1999). Review of methods for solving the eeg inverse problem. Int. J. Bioelectromagn. 1, 75–86.

[B198] Penney E. S. Abbott M. J. (2014). Anticipatory and post-event rumination in social anxiety disorder: a review of the theoretical and empirical literature. Behav. Change 31, 79–101. 10.1017/bec.2014.330886898

[B199] Petrides M. (2005). Lateral prefrontal cortex: architectonic and functional organization. Philos. Trans. R. Soc. B Biol. Sci. 360, 781–795. 10.1098/rstb.2005.1631 15937012 PMC1569489

[B200] Pfeiffer U. J. Timmermans B. Vogeley K. Frith C. Schilbach L. (2013). Towards a neuroscience of social interaction. Front. Human Neurosci. 7:22. 10.3389/fnhum.2013.00022 23378836 PMC3561599

[B201] Phillips M. L. Ladouceur C. D. Drevets W. C. (2008). A neural model of voluntary and automatic emotion regulation: implications for understanding the pathophysiology and neurodevelopment of bipolar disorder. Mol. Psychiatry 13, 833–857. 10.1038/mp.2008.65 18574483 PMC2745893

[B202] Picard R. W. (2000). Affective Computing (MIT press). p. 243

[B203] Prpa M. Pasquier P. (2019). Brain-computer interfaces in contemporary art: a state of the art and taxonomy, in Brain Art, ed A. Nijholt (Springer), 65–115. 10.1007/978-3-030-14323-7

[B204] Qiu C. Liao W. Ding J. Feng Y. Zhu C. Nie X. Zhang W. Chen H. Gong Q. (2011). Regional homogeneity changes in social anxiety disorder: a resting-state fmri study. Psychiatry Res. Neuroimag. 194, 47–53. 10.1016/j.pscychresns.2011.01.010 21831605

[B205] Quaedflieg C. W. Smulders F. T. Meyer T. Peeters F. Merckelbach H. Smeets T. (2016). The validity of individual frontal alpha asymmetry eeg neurofeedback. Soc. Cogn. Affect. Neurosci. 11, 33–43. 10.1093/scan/nsv090 26163671 PMC4692315

[B206] Rapee R. M. Heimberg R. G. (1997). A cognitive-behavioral model of anxiety in social phobia. Behavi. Res. Therapy 35, 741–756. 10.1016/s0005-7967(97)00022-3 9256517

[B207] Raymond J. Varney C. Parkinson L. A. Gruzelier J. H. (2005). The effects of alpha/theta neurofeedback on personality and mood. Cogn. Brain Res. 23, 287–292. 10.1016/j.cogbrainres.2004.10.023 15820636

[B208] Roberts G. Holmes N. Alexander N. Boto E. Leggett J. Hill R. M.. (2019). Towards opm-meg in a virtual reality environment. Neuroimage 199, 408–417. 10.1016/j.neuroimage.2019.06.010 31173906 PMC8276767

[B209] Rodebaugh T. L. Holaway R. M. Heimberg R. G. (2004). The treatment of social anxiety disorder. Clin. Psychol. Rev. 24, 883–908. 10.1016/j.cpr.2004.07.007 15501560

[B210] Ros T. Enriquez-Geppert S. Zotev V. Young K. D. Wood G. Whitfield-Gabrieli S.. (2020). Consensus on the reporting and experimental design of clinical and cognitive-behavioural neurofeedback studies (cred-nf checklist). Brain 143, 1674–1685. 10.1093/brain/awaa009 32176800 PMC7296848

[B211] Rosenfeld J. P. Cha G. Blair T. Gotlib I. H. (1995). Operant (biofeedback) control of left-right frontal alpha power differences: potential neurotherapy for affective disorders. Biofeedback Self Regul. 20, 241–258. 10.1007/BF01474516 7495918

[B212] Rubinov M. Sporns O. (2010). Complex network measures of brain connectivity: uses and interpretations. Neuroimage 52, 1059–1069. 10.1016/j.neuroimage.2009.10.003 19819337

[B213] Rule R. R. Shimamura A. P. Knight R. T. (2002). Orbitofrontal cortex and dynamic filtering of emotional stimuli. Cogn. Affect. Behav. Neurosci. 2, 264–270. 10.3758/cabn.2.3.264 12775190

[B214] Saito D. N. Tanabe H. C. Izuma K. Hayashi M. J. Morito Y. Komeda H.. (2010). ‘stay tuned': inter-individual neural synchronization during mutual gaze and joint attention. Front. Integr. Neurosci. 4:127. 10.3389/fnint.2010.00127 21119770 PMC2990457

[B215] Salimpour Y. Anderson W. S. (2019). Cross-frequency coupling based neuromodulation for treating neurological disorders. Front. Neurosci. 13:125. 10.3389/fnins.2019.00125 30846925 PMC6393401

[B216] Sander T. Bock A. Leistner S. Kühn A. Trahms L. (2010). Coherence and imaginary part of coherency identifies cortico-muscular and cortico-thalamic coupling, in 2010 Annual International Conference of the IEEE Engineering in Medicine and Biology (Buenos Aires: IEEE), 1714–1717. 10.1109/IEMBS.2010.5626851 21096404

[B217] Sänger J. Müller V. Lindenberger U. (2012). Intra-and interbrain synchronization and network properties when playing guitar in duets. Front. Human Neurosci. 6:312. 10.3389/fnhum.2012.00312 23226120 PMC3509332

[B218] Sarkheil P. Zilverstand A. Kilian-Hütten N. Schneider F. Goebel R. Mathiak K. (2015). fmri feedback enhances emotion regulation as evidenced by a reduced amygdala response. Behav. Brain Res. 281, 326–332. 10.1016/j.bbr.2014.11.027 25461265

[B219] Schelter B. Timmer J. Eichler M. (2009). Assessing the strength of directed influences among neural signals using renormalized partial directed coherence. J. Neurosci. Methods 179, 121–130. 10.1016/j.jneumeth.2009.01.006 19428518

[B220] Schilbach L. Timmermans B. Reddy V. Costall A. Bente G. Schlicht T.. (2013). Toward a second-person neuroscience. Behav. Brain Sci. 36, 393–414. 10.1017/S0140525X12000660 23883742

[B221] Schmidt L. A. (1999). Frontal brain electrical activity in shyness and sociability. Psychol. Sci. 10, 316–320. 10.1111/1467-9280.001617873702

[B222] Schutter D. J. Knyazev G. G. (2012). Cross-frequency coupling of brain oscillations in studying motivation and emotion. Motivat. Emotion 36, 46–54. 10.1007/s11031-011-9237-6 22448078 PMC3294206

[B223] Schutter D. J. Van Honk E. (2005). Salivary cortisol levels and the coupling of midfrontal delta-beta oscillations. Int. J. Psychophysiol. 55, 127–129. 10.1016/j.ijpsycho.2004.07.003 15598522

[B224] Sekihara K. Owen J. P. Trisno S. Nagarajan S. S. (2011). Removal of spurious coherence in meg source-space coherence analysis. IEEE Trans. Biomed. Eng. 58, 3121–3129. 10.1109/TBME.2011.2162514 21824842 PMC4096348

[B225] Seth A. K. (2005). Causal connectivity of evolved neural networks during behavior. Netw. Comput. Neural Syst. 16, 35–54. 10.1080/09548980500238756 16350433

[B226] Seth A. K. Edelman G. M. (2007). Distinguishing causal interactions in neural populations. Neural Comput. 19, 910–933. 10.1162/neco.2007.19.4.910 17348767

[B227] Shah S. G. Klumpp H. Angstadt M. Nathan P. J. Phan K. L. (2009). Amygdala and insula response to emotional images in patients with generalized social anxiety disorder. J Psychiatry Neurosci. 34, 296–302. 19568481 PMC2702447

[B228] Shteynberg G. (2015). Shared attention. Perspect. Psychol. Sci. 10, 579–590. 10.1177/1745691615589104 26385997

[B229] Shteynberg G. (2018). A collective perspective: shared attention and the mind. Curr. Opin. Psychol. 23, 93–97. 10.1016/j.copsyc.2017.12.007 29317182

[B230] Siegel D. M. Burke T. A. Hamilton J. L. Piccirillo M. L. Scharff A. Alloy L. B. (2018). Social anxiety and interpersonal stress generation: the moderating role of interpersonal distress. Anxiety Stress Coping 31, 526–538. 10.1080/10615806.2018.1482723 29855206 PMC6344932

[B231] Sinha N. Maszczyk T. Wanxuan Z. Tan J. Dauwels J. (2016). Eeg hyperscanning study of inter-brain synchrony during cooperative and competitive interaction, in 2016 IEEE International Conference on Systems, Man, and Cybernetics (SMC) (Budapest). 10.1109/SMC.2016.7844990

[B232] Sladky R. Höflich A. Atanelov J. Kraus C. Baldinger P. Moser E.. (2012). Increased neural habituation in the amygdala and orbitofrontal cortex in social anxiety disorder revealed by fmri. PLoS ONE 7:e50050. 10.1371/journal.pone.0050050 23209643 PMC3510234

[B233] Smith M. L. Collura T. F. Ferrera J. de Vries J. (2014). Infra-slow fluctuation training in clinical practice: a technical history. Neuroregulation 1, 187–187. 10.15540/nr.1.2.187

[B234] Soral W. Kofta M. Bukowski M. (2021). Helplessness experience and intentional (un-) binding: control deprivation disrupts the implicit sense of agency. J. Exp. Psychol. Gen. 150, 289. 10.1037/xge0000791 32658528

[B235] Spence S. H. Rapee R. M. (2016). The etiology of social anxiety disorder: an evidence-based model. Behav. Res. Therapy 86, 50–67. 10.1016/j.brat.2016.06.007 27406470

[B236] Spielberger C. (1983). State-Trait Anxiety Inventory for Adults (Palo Alto, CA). 10.1037/t06496-000

[B237] Sripada C. S. Angstadt M. Banks S. Nathan P. J. Liberzon I. Phan K. L. (2009). Functional neuroimaging of mentalizing during the trust game in social anxiety disorder. Neuroreport 20, 984. 10.1097/WNR.0b013e32832d0a67 19521264 PMC2746411

[B238] Stein D. J. Lim C. C. Roest A. M. De Jonge P. Aguilar-Gaxiola S. Al-Hamzawi A.. (2017). The cross-national epidemiology of social anxiety disorder: data from the world mental health survey initiative. BMC Med. 15, 1–21. 10.1186/s12916-017-0889-2 28756776 PMC5535284

[B239] Stein M. B. Stein D. J. (2008). Social anxiety disorder. 371, 1115–1125. 10.1016/S0140-6736(08)60488-2 18374843

[B240] Symons A. E. El-Deredy W. Schwartze M. Kotz S. A. (2016). The functional role of neural oscillations in non-verbal emotional communication. Front. Human Neurosci. 10:239. 10.3389/fnhum.2016.00239 27252638 PMC4879141

[B241] Szymanski C. Müller V. Brick T. R. von Oertzen T. Lindenberger U. (2017). Hyper-transcranial alternating current stimulation: experimental manipulation of inter-brain synchrony. Front. Human Neurosci. 11:539. 10.3389/fnhum.2017.00539 29167638 PMC5682643

[B242] Tanabe H. C. Kosaka H. Saito D. N. Koike T. Hayashi M. J. Izuma K.. (2012). Hard to “tune in”: neural mechanisms of live face-to-face interaction with high-functioning autistic spectrum disorder. Front. Human Neurosci. 6:268. 10.3389/fnhum.2012.00268 23060772 PMC3459004

[B243] Thompson M. Thompson L. (2003). The neurofeedback book: An introduction to basic concepts in applied psychophysiology, in Association for Applied Psychophysiology and Biofeedback (Colorado, CL: Association for Applied Psychophysiology and Biofeedback).

[B244] Tillfors M. Furmark T. Marteinsdottir I. Fredrikson M. (2002). Cerebral blood flow during anticipation of public speaking in social phobia: a pet study. Biol. Psychiatry 52, 1113–1119. 10.1016/s0006-3223(02)01396-3 12460694

[B245] Tognoli E. Lagarde J. DeGuzman G. C. Kelso J. S. (2007). The phi complex as a neuromarker of human social coordination. Proc. Natl. Acad. Sci. U.S.A. 104, 8190–8195. 10.1073/pnas.0611453104 17470821 PMC1859993

[B246] Tomarkenand A. J. Keener A. D. (1998). Frontal brain asymmetry and depression: a self-regulatory perspective. Cogn. Emotion 12, 387–420. 10.1080/026999398379655

[B247] Tomasello M. Akhtar N. (1995). Two-year-olds use pragmatic cues to differentiate reference to objects and actions. Cogn. Develop. 10, 201–224. 10.1016/0885-2014(95)90009-8

[B248] Tomasello M. Melis A. P. Tennie C. Wyman E. Herrmann E. Gilby I. C.. (2012). Two key steps in the evolution of human cooperation: the interdependence hypothesis. Curr. Anthropol. 53, 673–692. 10.1086/668207

[B249] Toppi J. Borghini G. Petti M. He E. J. De Giusti V. He B.. (2016). Investigating cooperative behavior in ecological settings: an eeg hyperscanning study. PLoS ONE 11:e0154236. 10.1371/journal.pone.0154236 27124558 PMC4849782

[B250] Tuscan L.-A. Herbert J. D. Forman E. M. Juarascio A. S. Izzetoglu M. Schultheis M. (2013). Exploring frontal asymmetry using functional near-infrared spectroscopy: a preliminary study of the effects of social anxiety during interaction and performance tasks. Brain Imag. Behav. 7, 140–153. 10.1007/s11682-012-9206-z 23132684

[B251] Uliaszek A. A. Zinbarg R. E. Mineka S. Craske M. G. Sutton J. M. Griffith J. W.. (2010). The role of neuroticism and extraversion in the stress–anxiety and stress–depression relationships. Anxiety Stress Coping 23, 363–381. 10.1080/10615800903377264 19890753 PMC3690955

[B252] Valdés P. Bosch J. Grave R. Hernandez J. Riera J. Pascual R. Biscay R. (1992). Frequency domain models of the eeg. Brain Topography 4, 309–319. 10.1007/BF01135568 1510874

[B253] Van Essen D. C. Ugurbil K. Auerbach E. Barch D. Behrens T. E. Bucholz R.. (2012). The human connectome project: a data acquisition perspective. Neuroimage 62, 2222–2231. 10.1016/j.neuroimage.2012.02.018 22366334 PMC3606888

[B254] Vernon D. Egner T. Cooper N. Compton T. Neilands C. Sheri A.. (2003). The effect of training distinct neurofeedback protocols on aspects of cognitive performance. Int. J. Psychophysiol. 47, 75–85. 10.1016/S0167-8760(02)00091-0 12543448

[B255] Viswesvaran C. Sanchez J. I. Fisher J. (1999). The role of social support in the process of work stress: A meta-analysis. J. Vocat. Behav. 54, 314–334. 10.1006/jvbe.1998.16619325800

[B256] Voelcker-Rehage C. Niemann C. Hübner L. Godde B. Winneke A. H. (2016). Benefits of physical activity and fitness for lifelong cognitive and motor development brain and behavior, in Sport and Exercise Psychology Research (Elsevier), 43–73.

[B257] Wang M.-Y. Luan P. Zhang J. Xiang Y.-T. Niu H. Yuan Z. (2018). Concurrent mapping of brain activation from multiple subjects during social interaction by hyperscanning: a mini-review. Quant. Imaging Med. Surgery 8, 819. 10.21037/qims.2018.09.07 30306062 PMC6177358

[B258] Watson D. Clark L. A. Tellegen A. (1988). Development and validation of brief measures of positive and negative affect: the panas scales. J. Personal. Soc. Psychol. 54, 1063–1070. 10.1037//0022-3514.54.6.1063 3397865

[B259] Wong Q. J. Rapee R. M. (2016). The aetiology and maintenance of social anxiety disorder: A synthesis of complementary theoretical models and formulation of a new integrated model. J. Affect. Disorders 203, 84–100. 10.1016/j.jad.2016.05.069 27280967

[B260] Yokoyama C. Kaiya H. Kumano H. Kinou M. Umekage T. Yasuda S.. (2015). Dysfunction of ventrolateral prefrontal cortex underlying social anxiety disorder: a multi-channel nirs study. Neuroimage Clin. 8, 455–461. 10.1016/j.nicl.2015.05.011 26106570 PMC4474365

[B261] Yun K. Watanabe K. Shimojo S. (2012). Interpersonal body and neural synchronization as a marker of implicit social interaction. Nature 2, 1–8. 10.1038/srep00959 23233878 PMC3518815

[B262] Zander T. O. Kothe C. Jatzev S. Gaertner M. (2010). Enhancing human-computer interaction with input from active and passive brain-computer interfaces, in Brain–Computer Interfaces. Human-Computer Interaction Series, eds Tan D. Nijholt A. (London: Springer), 181–199. 10.1007/978-1-84996-272-811

[B263] Zhang G. Jin Q. Lin M. (2005). A framework of social interaction support for ubiquitous learning, in 19th International Conference on Advanced Information Networking and Applications (AINA' 05), Vol. 1. (Taipei), 639–643. 10.1109/AINA.2005.2623252731

[B264] Zhang P. Cheng L. (2017). A randomized controlled trial of a neurofeedback-based training for improvement in social phobia disorder. NeuroQuantology 15, 133–138. 10.14704/nq.2017.15.4.1136

[B265] Zhang R. Zhao X. (2018). A cross-brain interaction platform based on neurofeedback using electroencephalogram, in International Conference on Augmented Cognition (Las Vegas, NV: Springer), 222–230.

[B266] Zhao Z. Yao S. Li K. Sindermann C. Zhou F. Zhao W.. (2019). Real-time functional connectivity-informed neurofeedback of amygdala-frontal pathways reduces anxiety. Psychotherapy Psychosomatics 88, 5–15. 10.1159/000496057 30699438

