# Corrigendum: A Two-Person Neuroscience Approach for Social Anxiety: A Paradigm With Interbrain Synchrony and Neurofeedback

**DOI:** 10.3389/fpsyg.2022.871022

**Published:** 2022-04-08

**Authors:** Marcia A. Saul, Xun He, Stuart Black, Fred Charles

**Affiliations:** ^1^Faculty of Media and Communication, Centre for Digital Entertainment, Bournemouth University, Poole, United Kingdom; ^2^Department of Psychology, Faculty of Science and Technology, Bournemouth University, Poole, United Kingdom; ^3^Applied Neuroscience Solutions Ltd., Frimley Green, United Kingdom; ^4^Department of Creative Technology, Faculty of Science and Technology, Bournemouth University, Poole, United Kingdom

**Keywords:** social anxiety disorder (SAD), hyperscanning, interbrain synchrony, neurofeedback, two-person neuroscience

In the original article, there was an error. In the “**Author Contributions**” section, all authors should have been assigned the same contributions.

A correction has been made to **Author Contributions**. The corrected statement is shown below.

## Author Contributions

MS, XH, SB, and FC have made substantial, direct, and intellectual contributions to the work and approved for publication. All authors contributed to the article and approved the submitted version.

The authors apologize for this error and state that this does not change the scientific conclusions of the article in any way. The original article has been updated.

## Publisher's Note

All claims expressed in this article are solely those of the authors and do not necessarily represent those of their affiliated organizations, or those of the publisher, the editors and the reviewers. Any product that may be evaluated in this article, or claim that may be made by its manufacturer, is not guaranteed or endorsed by the publisher.

